# ‘It depends’: what 86 systematic reviews tell us about what strategies to use to support the use of research in clinical practice

**DOI:** 10.1186/s13012-024-01337-z

**Published:** 2024-02-19

**Authors:** Annette Boaz, Juan Baeza, Alec Fraser, Erik Persson

**Affiliations:** 1 Health and Social Care Workforce Research Unit, The Policy Institute, King’s College London, Virginia Woolf Building, 22 Kingsway, London, WC2B 6LE UK; 2 King’s Business School, King’s College London, 30 Aldwych, London, WC2B 4BG UK; 3 Federal University of Santa Catarina (UFSC), Campus Universitário Reitor João Davi Ferreira Lima, Florianópolis, SC 88.040-900 Brazil

**Keywords:** Systematic review, Implementation, Strategies, Interventions, Clinical practice, Research evidence, Single, Multi-faceted

## Abstract

**Background:**

The gap between research findings and clinical practice is well documented and a range of strategies have been developed to support the implementation of research into clinical practice. The objective of this study was to update and extend two previous reviews of systematic reviews of strategies designed to implement research evidence into clinical practice.

**Methods:**

We developed a comprehensive systematic literature search strategy based on the terms used in the previous reviews to identify studies that looked explicitly at interventions designed to turn research evidence into practice. The search was performed in June 2022 in four electronic databases: Medline, Embase, Cochrane and Epistemonikos. We searched from January 2010 up to June 2022 and applied no language restrictions. Two independent reviewers appraised the quality of included studies using a quality assessment checklist. To reduce the risk of bias, papers were excluded following discussion between all members of the team. Data were synthesised using descriptive and narrative techniques to identify themes and patterns linked to intervention strategies, targeted behaviours, study settings and study outcomes.

**Results:**

We identified 32 reviews conducted between 2010 and 2022. The reviews are mainly of multi-faceted interventions (*n* = 20) although there are reviews focusing on single strategies (ICT, educational, reminders, local opinion leaders, audit and feedback, social media and toolkits). The majority of reviews report strategies achieving small impacts (normally on processes of care). There is much less evidence that these strategies have shifted patient outcomes. Furthermore, a lot of nuance lies behind these headline findings, and this is increasingly commented upon in the reviews themselves.

**Discussion:**

Combined with the two previous reviews, 86 systematic reviews of strategies to increase the implementation of research into clinical practice have been identified. We need to shift the emphasis away from isolating individual and multi-faceted interventions to better understanding and building more situated, relational and organisational capability to support the use of research in clinical practice. This will involve drawing on a wider range of research perspectives (including social science) in primary studies and diversifying the types of synthesis undertaken to include approaches such as realist synthesis which facilitate exploration of the context in which strategies are employed.

**Supplementary Information:**

The online version contains supplementary material available at 10.1186/s13012-024-01337-z.

Contribution to the literature

- Considerable time and money is invested in implementing and evaluating strategies to increase the implementation of research into clinical practice.
- The growing body of evidence is not providing the anticipated clear lessons to support improved implementation.
- Instead what is needed is better understanding and building more situated, relational and organisational capability to support the use of research in clinical practice.
- This would involve a more central role in implementation science for a wider range of perspectives, especially from the social, economic, political and behavioural sciences and for greater use of different types of synthesis, such as realist synthesis.

## Introduction

The gap between research findings and clinical practice is well documented and a range of interventions has been developed to increase the implementation of research into clinical practice [1, 2]. In recent years researchers have worked to improve the consistency in the ways in which these interventions (often called strategies) are described to support their evaluation. One notable development has been the emergence of Implementation Science as a field focusing explicitly on “the scientific study of methods to promote the systematic uptake of research findings and other evidence-based practices into routine practice” ([3] p. 1). The work of implementation science focuses on closing, or at least narrowing, the gap between research and practice. One contribution has been to map existing interventions, identifying 73 discreet strategies to support research implementation [4] which have been grouped into 9 clusters [5]. The authors note that they have not considered the evidence of effectiveness of the individual strategies and that a next step is to understand better which strategies perform best in which combinations and for what purposes [4]. Other authors have noted that there is also scope to learn more from other related fields of study such as policy implementation [6] and to draw on methods designed to support the evaluation of complex interventions [7].

The increase in activity designed to support the implementation of research into practice and improvements in reporting provided the impetus for an update of a review of systematic reviews of the effectiveness of interventions designed to support the use of research in clinical practice [8] which was itself an update of the review conducted by Grimshaw and colleagues in 2001. The 2001 review [9] identified 41 reviews considering a range of strategies including educational interventions, audit and feedback, computerised decision support to financial incentives and combined interventions. The authors concluded that all the interventions had the potential to promote the uptake of evidence in practice, although no one intervention seemed to be more effective than the others in all settings. They concluded that combined interventions were more likely to be effective than single interventions. The 2011 review identified a further 13 systematic reviews containing 313 discrete primary studies. Consistent with the previous review, four main strategy types were identified: audit and feedback; computerised decision support; opinion leaders; and multi-faceted interventions (MFIs). Nine of the reviews reported on MFIs. The review highlighted the small effects of single interventions such as audit and feedback, computerised decision support and opinion leaders. MFIs claimed an improvement in effectiveness over single interventions, although effect sizes remained small to moderate and this improvement in effectiveness relating to MFIs has been questioned in a subsequent review [10]. In updating the review, we anticipated a larger pool of reviews and an opportunity to consolidate learning from more recent systematic reviews of interventions.

## Methods

This review updates and extends our previous review of systematic reviews of interventions designed to implement research evidence into clinical practice. To identify potentially relevant peer-reviewed research papers, we developed a comprehensive systematic literature search strategy based on the terms used in the Grimshaw et al. [9] and Boaz, Baeza and Fraser [8] overview articles. To ensure optimal retrieval, our search strategy was refined with support from an expert university librarian, considering the ongoing improvements in the development of search filters for systematic reviews since our first review [11]. We also wanted to include technology-related terms (e.g. apps, algorithms, machine learning, artificial intelligence) to find studies that explored interventions based on the use of technological innovations as mechanistic tools for increasing the use of evidence into practice (see Additional file 1: Appendix A for full search strategy).

The search was performed in June 2022 in the following electronic databases: Medline, Embase, Cochrane and Epistemonikos. We searched for articles published since the 2011 review. We searched from January 2010 up to June 2022 and applied no language restrictions. Reference lists of relevant papers were also examined.

We uploaded the results using EPPI-Reviewer, a web-based tool that facilitated semi-automation of the screening process and removal of duplicate studies. We made particular use of a priority screening function to reduce screening workload and avoid ‘data deluge’ [12]. Through machine learning, one reviewer screened a smaller number of records (*n* = 1200) to train the software to predict whether a given record was more likely to be relevant or irrelevant, thus pulling the relevant studies towards the beginning of the screening process. This automation did not replace manual work but helped the reviewer to identify eligible studies more quickly. During the selection process, we included studies that looked explicitly at interventions designed to turn research evidence into practice. Studies were included if they met the following pre-determined inclusion criteria:

- The study was a systematic review
- Search terms were included
- Focused on the implementation of research evidence into practice
- The methodological quality of the included studies was assessed as part of the review

Study populations included healthcare providers and patients. The EPOC taxonomy [13] was used to categorise the strategies. The EPOC taxonomy has four domains: delivery arrangements, financial arrangements, governance arrangements and implementation strategies. The implementation strategies domain includes 20 strategies targeted at healthcare workers. Numerous EPOC strategies were assessed in the review including educational strategies, local opinion leaders, reminders, ICT-focused approaches and audit and feedback. Some strategies that did not fit easily within the EPOC categories were also included. These were social media strategies and toolkits, and multi-faceted interventions (MFIs) (see Table 2). Some systematic reviews included comparisons of different interventions while other reviews compared one type of intervention against a control group. Outcomes related to improvements in health care processes or patient well-being. Numerous individual study types (RCT, CCT, BA, ITS) were included within the systematic reviews.

We excluded papers that:

- Focused on changing patient rather than provider behaviour
- Had no demonstrable outcomes
- Made unclear or no reference to research evidence

The last of these criteria was sometimes difficult to judge, and there was considerable discussion amongst the research team as to whether the link between research evidence and practice was sufficiently explicit in the interventions analysed. As we discussed in the previous review [8] in the field of healthcare, the principle of evidence-based practice is widely acknowledged and tools to change behaviour such as guidelines are often seen to be an implicit codification of evidence, despite the fact that this is not always the case.

**Fig. 1:**
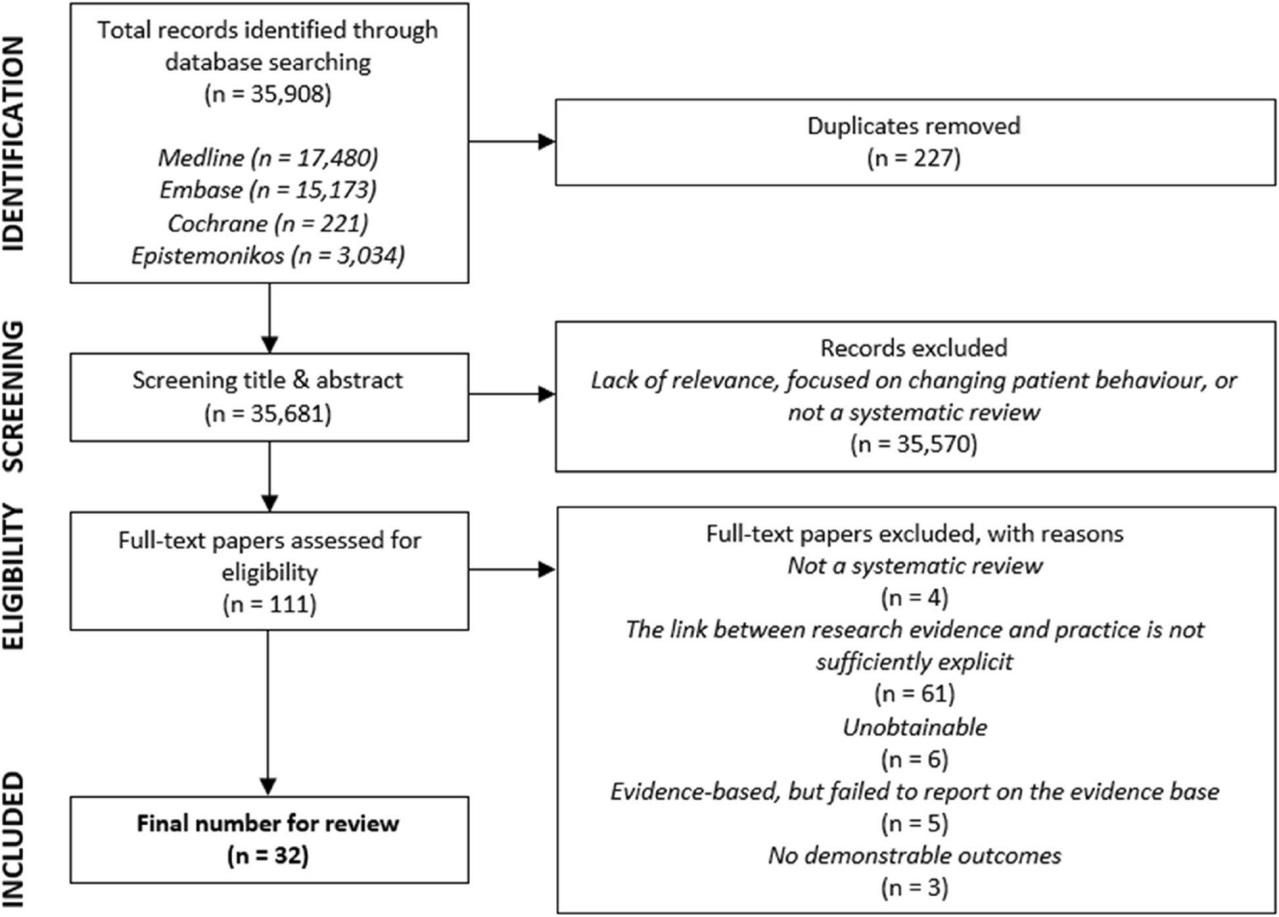
PRISMA flow diagram. Source: authors

Reviewers employed a two-stage process to select papers for inclusion. First, all titles and abstracts were screened by one reviewer to determine whether the study met the inclusion criteria. Two papers [14, 15] were identified that fell just before the 2010 cut-off. As they were not identified in the searches for the first review [8] they were included and progressed to assessment. Each paper was rated as include, exclude or maybe. The full texts of 111 relevant papers were assessed independently by at least two authors. To reduce the risk of bias, papers were excluded following discussion between all members of the team. 32 papers met the inclusion criteria and proceeded to data extraction. The study selection procedure is documented in a PRISMA literature flow diagram (see Fig. 1). We were able to include French, Spanish and Portuguese papers in the selection reflecting the language skills in the study team, but none of the papers identified met the inclusion criteria. Other non- English language papers were excluded.

One reviewer extracted data on strategy type, number of included studies, local, target population, effectiveness and scope of impact from the included studies. Two reviewers then independently read each paper and noted key findings and broad themes of interest which were then discussed amongst the wider authorial team. Two independent reviewers appraised the quality of included studies using a Quality Assessment Checklist based on Oxman and Guyatt [16] and Francke et al. [17]. Each study was rated a quality score ranging from 1 (extensive flaws) to 7 (minimal flaws) (see Additional file 2: Appendix B). All disagreements were resolved through discussion. Studies were not excluded in this updated overview based on methodological quality as we aimed to reflect the full extent of current research into this topic.

The extracted data were synthesised using descriptive and narrative techniques to identify themes and patterns in the data linked to intervention strategies, targeted behaviours, study settings and study outcomes.

## Results

Thirty-two studies were included in the systematic review. Table 1. provides a detailed overview of the included systematic reviews comprising reference, strategy type, quality score, number of included studies, local, target population, effectiveness and scope of impact (see Table 1. at the end of the manuscript). Overall, the quality of the studies was high. Twenty-three studies scored 7, six studies scored 6, one study scored 5, one study scored 4 and one study scored 3. The primary focus of the review was on reviews of effectiveness studies, but a small number of reviews did include data from a wider range of methods including qualitative studies which added to the analysis in the papers [18–21]. The majority of reviews report strategies achieving small impacts (normally on processes of care). There is much less evidence that these strategies have shifted patient outcomes. In this section, we discuss the different EPOC-defined implementation strategies in turn. Interestingly, we found only two ‘new’ approaches in this review that did not fit into the existing EPOC approaches. These are a review focused on the use of social media and a review considering toolkits. In addition to single interventions, we also discuss multi-faceted interventions. These were the most common intervention approach overall. A summary is provided in Table 2.

**Table 1:** Characteristics and results of included systematic reviews

**Strategy type**	**Study reference**	**Systematic review quality score**	**Number of included studies**	**Context: setting and target population**	**Conclusions: effectiveness, scope of impact, quality of individual studies**
**Educational strategies**	**Grudniewicz A. et al.** *What is the effectiveness of printed educational materials on primary care physician knowledge, behaviour, and patient outcomes: a systematic review and meta-analyses* (2015)	7	40	**Setting:** High income countries (HIC)	**Effectiveness:** No significant effect was found on clinically important patient outcomes, physician behaviour, or physician cognition when printed educational materials (PEMs) were compared to usual care.
				**Target population:** primary care physicians (PCPs)	**Scope of impact:** PEMs were not effective at improving patient outcomes, knowledge, or behaviour of PCPs.
					**Quality of individual studies:** The reported quality of evidence ranged from low to high and many studies were unclear on important methodological factors. Using the Cochrane Effective Practice and Organization of Care (EPOC) risk of bias assessment tool, 33 Randomised Controlled Trials (RCTs) had unclear or high risk of bias for at least two criteria. Two RCTs were appraised as low risk of bias on eight of nine criteria, and only two RCTs were appraised as having low risk of bias on all nine criteria. The ITS studies had at least two unclear risk of bias out of seven criteria.
**Educational strategies**	**Koota E., Kääriäinen M. & Melender H-L.** *Educational interventions promoting evidence-based practice among emergency nurses: A systematic review* (2018)	7	10	**Setting:** unclear/not specified	**Effectiveness:** Interventions involving face-to-face contact led to significant or highly significant effects on patient benefits and emergency nurses’ knowledge, skills, and behavior. Interventions using written self-directed learning material led to significant improvements in nurses’ knowledge of evidence based practice (EBP).
				**Target population:** emergency nurses	**Scope of impact:** Most of the studied interventions had promising effects on emergency nurses’ EBP. The review primarily included small studies with low response rates, and many of them relied on self-assessed outcomes. The strength of the evidence for these outcomes is modest.
					**Quality of individual studies:** Three of the ten original studies were considered to be of excellent quality.
**Educational strategies**	**Wu Y. et al.** *Do educational interventions aimed at nurses to support the implementation of evidence-based practice improve patient outcomes? A systematic review* (2018)	7	18	**Setting:** HIC	**Effectiveness:** Although based on evaluation projects and qualitative data, the results suggest that positive changes on patient outcomes can be made following the implementation of specific evidence-based approaches (or projects). Four studies stated that conducting EBP projects in clinical practice improved patient outcomes but did not report specific details. Furthermore, one study reported mixed results regarding patient outcomes.
				**Target population:** nurses	**Scope of impact:** This review provided evidence that conducting EBP educational interventions on nurses in clinical practice can have a positive impact on patient outcomes, which can demonstrate the usefulness and importance of such programmes.
					**Quality of individual studies:** The quality of the 18 studies was assessed using the HCPRDU (Health Care Practice R&D Unit) evaluation tools and the scoring system developed for the purpose of this review. Five of the studies were assessed as excellent, seven studies could be classified as having some limitations, and six as having many limitations.
**Local opinion leaders**	**Flodgren G. et al.** *Local opinion leaders: effects on professional practice and healthcare outcomes* (2019)	7	24	**Setting:** HIC	**Effectiveness:** Local opinion leaders alone, or in combination with other interventions, can be effective in promoting evidence‐based practice, but the effectiveness varies both within and between studies. The effect on patient outcomes is uncertain. The review found that, overall, any intervention involving opinion leaders (OLs) probably improves healthcare professionals' compliance with evidence-based practice. The effect, however, varies within and across studies.
				**Target population:** health care professionals	**Scope of impact:** The use of OLs probably improves the ability of healthcare professionals to follow evidence-based guidelines, but we do not know if patient outcomes are improved.
					**Quality of individual studies:** All included studies were randomised and were initially considered to have a high certainty of evidence (before assessment of quality). The certainty of evidence for the main outcome (compliance with evidence-based practice) was downgraded from high to moderate certainty of evidence due to high risk of bias.
**Reminders**	**Arditi C. et al.** *Computer-generated reminders delivered on paper to healthcare professionals: effects on professional practice and healthcare outcomes* (2017)	7	34	**Setting:** HIC/low and middle income countries (LMIC)	**Effectiveness:** There is moderate-certainty evidence that computer-generated reminders delivered on paper to healthcare professionals probably slightly improves quality of care, in terms of compliance with preventive guidelines and compliance with disease management guidelines. It is uncertain whether reminders improve patient outcomes because the certainty of the evidence is very low. The heterogeneity of the reminder interventions included in this review also suggests that reminders can probably improve quality of care in various settings under various conditions. The authors are uncertain whether reminders, alone or in addition to co-intervention(s), improve patient outcomes as the certainty of the evidence is very low.
				**Target population:** healthcare professionals	**Scope of impact:** Providing reminders to doctors probably improves slightly the quality of care patients receive. However, because the certainty of the evidence is moderate, more high-quality studies on the effectiveness of reminders are needed to confirm to findings of this review.
					**Quality of individual studies:** The quality of the studies was fairly low. One reason for the low quality of studies was that reporting of earlier studies was very poor, thus making it difficult to assess whether appropriate measures were taken to reduce bias.
**Reminders**	**Pantoja T. et al.** *Manually-generated reminders delivered on paper: effects on professional practice and patient outcomes* (2019)	7	63	**Setting:** HIC/LMIC	**Effectiveness:** Manually-generated reminders delivered on paper as a single intervention probably lead from small to moderate increases in outcomes related to adherence to clinical recommendations, and they could be used as a single quality improvement (QI) intervention.
				**Target population:** health care professionals	**Scope of impact:** Forty-eight studies reported changes in professional practice measured as dichotomous process adherence outcomes (e.g., compliance with guidelines recommendations), 16 reported those changes measured as continuous process-of-care outcomes (e.g., number of days with catheters), eight reported dichotomous patient outcomes (e.g., mortality rates) and five reported continuous patient outcomes (e.g., mean systolic blood pressure).
					**Quality of individual studies:** Only one study was judged to be at high risk of reporting bias, with 41 studies assessed as low risk. The remaining 21 Studies were rated at unclear risk of selective reporting.
**ICT focused approaches**	**De Angelis G. et al.** *Information and communication technologies for the dissemination of clinical practice guidelines to health professionals: a systematic review* (2016)	6	21	**Setting:** unclear/not specified	**Effectiveness:** Website studies demonstrated significant improvements in perceived usefulness and perceived ease of use, but not for knowledge, reducing barriers, and intention to use clinical practice guidelines. Computer software studies demonstrated significant improvements in perceived usefulness, but not for knowledge and skills. Web-based workshop and email studies demonstrated significant improvements in knowledge, perceived usefulness, and skills. An electronic educational game intervention demonstrated a significant improvement from baseline in knowledge after 12 and 24 weeks. Computerized decision support system studies demonstrated variable findings for improvement in skills. Multifaceted interventions demonstrated significant improvements in beliefs about capabilities, perceived usefulness, and intention to use clinical practice guidelines, but variable findings for improvements in skills.
				**Target population:** health professionals (eg, physicians including medical residents, nurses, and physiotherapists)	**Scope of impact:** While the evidence is limited, studies of information and communication technologies (ICTs) included in this review have shown promising findings. ICTs are novel ways of disseminating clinical practice guidelines (CPGs), compared with more traditional methods. This review highlights which ICTs have been successfully used as a dissemination strategy for CPGs; however, it remains unclear whether one ICT is more effective than another. It is also unclear whether other ICTs not captured in this review, such as social media, can be used as effective dissemination strategies for CPGs.
					**Quality of individual studies:** The overall methodological quality of included studies was strong for the website studies, while it was uncertain for the electronic education game, email, and multifaceted studies.
**ICT focused approaches**	**Brown A. et al.** *Effectiveness of technology-enabled knowledge translation strategies in improving the use of research in public health: systematic review* (2020)	7	8	**Setting:** HIC	**Effectiveness:** Digital technology-enabled knowledge translation (TEKT) interventions may be effective at improving public health professionals’ knowledge, and may be as effective at improving knowledge as a face-to-face knowledge translation (KT) approach. The findings suggest that although a digital TEKT intervention may improve knowledge, the effects of such interventions on other outcomes are equivocal.
				**Target population:** health professionals, including nurses, child care health consultants, physiotherapists, primary health care workers, and public health practitioners	**Scope of impact:** The effectiveness of digital TEKT strategies relative to control or other KT interventions for self-efficacy or behavioral intention outcomes and changes to health policy or practice were mixed. Such findings offer little guidance for those interested in utilizing digital TEKT strategies to promote the transfer of knowledge to improve public health and demonstrate a considerable need for further research in this field.
					**Quality of individual studies:** Of the 8 studies, 7 were assessed as having an overall high risk of bias. All 8 studies were classified as having some concerns in relation to the selection of the reported results.
**ICT focused approaches**	**Jamal A., McKenzie K. & Clark M.** *The impact of health information technology on the quality of medical and health care: a systematic review* (2009)	3	23	**Setting:** HIC	**Effectiveness:** A positive improvement, in relation to their compliance with evidence-based guidelines, was seen in 14 studies. Studies that included an assessment of patient outcomes, however, showed insufficient evidence of either clinically or statistically important improvements
				**Target population:** clinicians	**Scope of impact:** In the current review, 14 out of 17 studies that assessed the impact of health information technology (HIT)/health information systems (HIS) on health care practitioners’ performance, revealed a positive improvement in relation to their compliance with evidence-based guidelines. The impact of HIS/HIT on the patient’s outcomes however inconsistent as only a small proportion of studies found benefits.
					**Quality of individual studies:** Owing to the fact that these study designs are prone to bias, the studies’ methodological quality was assessed with caution. For this purpose, a standardised quality assessment checklist for non-randomised studies was used, obtained from the Joanna-Briggs Institute. Therefore, only studies that met the minimum criteria as outlined in the quality assessment checklist were included for final review.
**Audit & feedback**	**Sykes M.J., McAnuff J. & Kolehmainen N.** *When is audit and feedback effective in dementia care? A systematic review* (2018)	4	13	**Setting:** unclear/not specified	**Effectiveness:** Included studies demonstrated large variation in the effectiveness of audit and feedback.
				**Target population:** health professionals	**Scope of impact:** All studies sought change in structures and processes; however, only one also sought change in outcome.
					**Quality of individual studies:** Methodological and reporting limitations in the included studies hinder the ability to draw strong conclusions on the effectiveness of audit and feedback in dementia care.
**Non-EPOC listed strategies: Social media**	**Bhatt N.R. et al.** *A systematic review of the use of social media for dissemination of clinical practice guidelines* (2021)	7	5	**Setting:** HIC	**Effectiveness:** The development of CPGs was driven by the principles of evidenced-based medicine, and that social media has the potential to improve CPGs dissemination. The papers shows that social media impact on CPG dissemination, knowledge and implementation. Methods of dissemination were highly variable. Two studies reported statistically significant improvement in knowledge, awareness, compliance, and positive behavior with respect to CPGs.
				**Target population:** clinicians	**Scope of impact:** The qualitative synthesis of the data and outcomes reported revealed a significant improvement in knowledge, aware- ness, compliance, and positive behavior with respect to CPGs with the use of social media dissemination compared with traditional methods of dissemination.
					**Quality of individual studies:** Overall, the studies were of average quality, considering the limitation of the lack of a comparison arm.
**Non-EPOC listed strategies: Toolkits**	**Yamada J. et al.** *The effectiveness of toolkits as knowledge translation strategies for integrating evidence into clinical care: a systematic review* (2015)	7	39	**Setting:** unclear/not specified	**Effectiveness:** Toolkits, either alone or as part of a multi-strategy intervention, hold promise as an effective approach for facilitating evidence use in practice and improving outcomes across a variety of disease states and healthcare settings. Six of the eight toolkits were partially or mostly effective in changing clinical outcomes and six studies reported on implementation outcomes. The types of resources embedded within toolkits varied but included predominantly educational materials.
				**Target population:** healthcare providers	**Scope of impact:** There was significant variation in the combination and type of KT strategies contained within the toolkits, a range of diseases for which they were developed, and a variety of intended knowledge users (e.g., health professionals or patients/caregivers), all of which contributed to key knowledge gaps.
					**Quality of individual studies:** The majority of studies (n=26) were rated as methodologically weak on the EPHPP tool (i.e., in terms of study design, selection bias, confounders, blinding, data collection methods and withdrawals and drop-outs); with 8 studies rated as moderate; and 5 as strong.
**Multi-faceted Interventions (MFIs)**	**Afari-Asiedu S. et al.** *Interventions to improve dispensing of antibiotics at the community level in low and middle income countries: a systematic review* (2022)	6	13	**Setting:** LMICs	**Effectiveness:** Educational meetings were the most frequently applied interventions and were effective in improving appropriate antibiotics dispensing. No evidence suggesting that multiple interventions were more effective than single interventions.
				**Target population:** community health posts (CHPs), over-the-counter medicine sellers (OTCMSs) and community pharmacies	**Scope of impact:** All studies reported positive effects following interventions to improve dispensing of medicines including antibiotics at the community level. Both providers and patient outcomes reported.
					**Quality of individual studies:** Trials/studies were generally rated as good quality and largely reported low risk of bias. Some observational studies were rated as poor-quality studies with high risk of bias.
**Multi-faceted Interventions (MFIs)**	**Boonacker C.W.B. et al.** *Interventions in health care professionals to improve treatment in children with upper respiratory tract infections* (2010)	6	10	**Setting:** unclear/not specified	**Effectiveness:** All strategies used (i.e., computer interventions, educational sessions with or without education materials, collaborative development of guidelines and a training video in combination with a risk factor checklist) were effective in changing health care professionals practice regarding children with URTIs. Multifaceted and computer strategies work best.
				**Target population:** health care professionals’ behavior in the management of children with upper respiratory tract infections (URTIs).	**Scope of impact:** Most strategies were aimed at changing antibiotic prescribing behavior in children with acute otitis media.
					**Quality of individual studies:** 17% of the quality criteria were judged as unclear since many studies provided not enough information to judge the risk of bias as no risk or high risk. Contamination may have played a role in three studies, which may have caused an underestimation of the effect of the interventions.
**Multi-faceted Interventions (MFIs)**	**Al Zoubi F.M. et al.** *The effectiveness of interventions designed to increase the uptake of clinical practice guidelines and best practices among musculoskeletal professionals: a systematic review* (2018)	7	11	**Setting:** HIC	**Effectiveness:** Findings suggest that for professional outcomes, single-component KT interventions are more effective than no intervention, and multifaceted interventions are more effective than single-component interventions. Single-component KT interventions such as interactive educational meetings and educational materials were found to have a small effect on enhancing professional adherence to clinical practice guidelines. Some studies that assessed multifaceted interventions reported mixed results.
				**Target population:** musculoskeletal professionals	**Scope of impact:** The findings suggested that multifaceted educational KT interventions appear to be effective for improving professional outcomes, although effects were inconsistent. The KT strategies were generally not effective on patient outcomes.
					**Quality of individual studies:** The majority of the included studies were considered to have moderate-to-high risk of bias. In general, studies were of low quality.
**Multi-faceted Interventions (MFIs)**	**Ariyo P. et al.** *Implementation strategies to reduce surgical site infections: a systematic review* (2019)	5	8	**Setting:** LMIC	**Effectiveness:** Multifaceted implementation strategies represent the most common approach to facilitating the adoption of evidence-based practices.
				**Target population:** health professionals	**Scope of impact:** Implementation strategies to improve adherence with evidence-based SSI-prevention interventions.
					**Quality of individual studies:** Study quality was appraised with the EPOC criteria. Only 8 studies met the EPOC criteria of an acceptable-quality study design.
**Multi-faceted Interventions (MFIs)**	**Borgert M.J., Goossens A. & Dongelmans D.A.** *What are effective strategies for the implementation of care bundles on ICUs: a systematic review* (2015)	7	47	**Setting:** unclear/not specified	**Effectiveness:** The three most frequently used strategies were education, reminders and audit and feedback. The authors could not identify the most effective implementation strategy that resulted in the highest levels of compliance.
				**Target population:** health providers in adult intensive care	**Scope of impact:** Care bundles have already proven to be effective in reducing negative clinical outcomes. It is important to mention that the authors focused on finding the best implementation strategy to achieve high levels of bundle compliance and not on the outcome of care processes.
					**Quality of individual studies:** 77% of the studies scored between 15 and 19 points on the Downs and Black quality assessment scale and were classified as fair. 13% of the studies scored 20 points or more and were classified as good. 11% of the studies were classified as poor. The authors assessed reporting bias of the included studies, and no studies were found reporting negative results.
**Multi-faceted Interventions (MFIs)**	**Cahill L. et al.** *Implementation interventions to promote the uptake of evidence- based practices in stroke rehabilitation (Review)* (2020)	7	9	**Setting:** HIC	**Effectiveness:** The authors could not obtain a reliable estimate of the effect of stroke rehabilitation implementation interventions on healthcare professional adherence to evidence-based practice compared with no intervention as the certainty of the evidence is very low.
				**Target population:** healthcare professionals in stroke rehabilitation	**Scope of impact:** Main outcomes were healthcare professional adherence to recommended treatment, patient adherence to recommended treatment, patient health status and well-being, healthcare professional intention and satisfaction, resource use outcomes and adverse effects.
					**Quality of individual studies:** The certainty of the evidence was considered very low.
**Multi-faceted Interventions (MFIs)**	**Pedersen E.R. et al.** *Elusive search for effective provider interventions: a systematic review of provider interventions to increase adherence to evidence-based treatment for depression* (2018)	7	22	**Setting:** unclear/not specified	**Effectiveness:** Findings provide little support for the effectiveness of currently tested provider education or dissemination interventions on provider adherence to depression treatment guidelines; however, there was some evidence that provider interventions improved the outcomes of medication prescribing and patient depression treatment response. Results also suggested that some interventions that were tailored to providers’ needs and that went beyond simply distributing guidelines to providers may improve provider behavior and promote guideline adherence.
				**Target population:** healthcare providers responsible for patient care in the outpatient setting	**Scope of impact:** Results were heterogeneous and analyses comparing provider interventions with usual clinical practice did not indicate a statistically significant difference in guideline adherence across studies. There was some evidence that provider interventions improved individual outcomes such as medication prescribing and indirect comparisons indicated more complex provider interventions may be associated with more favorable outcomes.
					**Quality of individual studies:** The methodological rigor of the included studies was variable; however, all studies were rated high risk of performance bias related to the lack of blinding of intervention providers.
**Multi-faceted Interventions (MFIs)**	**Jenkins H.J. et al.** *Effectiveness of interventions designed to reduce the use of imaging for low-back pain: a systematic review* (2015)	6	7	**Setting:** unclear/not specified	**Effectiveness:** Clinical decision support in a hospital setting and targeted reminders to pri­mary care doctors were effective interventions in reducing the use of imaging for low-back pain.
				**Target population:** healthcare professionals	**Scope of impact:** Clinical decision support involving a modified referral form in a hospital setting and targeted reminders to primary care doctors of appropriate indications for imaging were interventions that significantly decreased the use of imaging for low-back pain by 36.8% and 22.5%, respectively. These strategies are potentially low-cost interventions that would substantially decrease medical expenditures associated with the management of low-back pain.
					**Quality of individual studies:** All of the RCTs reported using adequate randomization and allocation procedures and objective outcome measures. However, they were unable to blind the practitioners involved in the study.
**Multi-faceted Interventions (MFIs)**	**Bennett S. et al.** *Implementation of evidence-based, non-pharmacological interventions addressing behavior and psychological symptoms of dementia: a systematic review focused on implementation strategies* (2021)	6	12	**Setting:** HIC	**Effectiveness:** Multiple implementation strategies to increase the use of nonpharmacological interventions have demonstrated clinical improvements when provided by clinicians as part of their everyday work routines.
				**Target population:** health professionals/care staff	**Scope of impact:** One of the key messages from this review is that implementation studies were able to demonstrate clinical improvements, despite substantial changes to original clinical interventions introduced to accommodate the constraints of clinical practice. Seven implementation studies reported positive (small, beneficial effects) outcomes for clients on some aspect of behavior or depression for the person with dementia. The majority of studies also reported some improvement in carers’ ability to respond to behavioral and psychological symptoms of dementia. However, this review did not set out to identify studies implementing interventions that had focused on carer outcomes.
					**Quality of individual studies:** Overall, methods and results were clearly reported.
**Multi-faceted Interventions (MFIs)**	**Noonan V.K. et al.** *Knowledge translation and implementation in spinal cord injury: a systematic review* (2014)	7	13	**Setting:** HIC	**Effectiveness:** All studies used multiple implementation drivers. KT interventions included developing and implementing patient care protocols, providing clinician education and incorporating outcome measures into clinical practice. The methods (or drivers) to facilitate the implementation included organizing training sessions for clinical staff, introducing computerized reminders and involving organizational leaders.
				**Target population:** clinicians	**Scope of impact:** There is some evidence that KT interventions may change clinician behavior and improve patient outcomes. 3 studies reported significant behavior change. Out of the 10 studies, 6 evaluated the effect of the implementation on patient outcomes using statistical analyses, with 4 reporting significant improvements.
					**Quality of individual studies:** The majority of the articles were considered to be poor, suggesting the need for more rigorous study methodology.
**Multi-faceted Interventions (MFIs)**	**Yost J. et al.** *The effectiveness of knowledge translation interventions for promoting evidence-informed decision-making among nurses in tertiary care: a systematic review and meta-analysis* (2015)	7	30	**Setting:** HIC	**Effectiveness:** Recommendations cannot be drawn about the relative effectiveness of single or multifaceted KT interventions or components of these interventions. No studies evaluated the impact on knowledge and skills; they primarily investigated the effectiveness of multifaceted KT strategies for promoting evidence informed decision-making (EIDM) behaviours and improving client outcomes. Almost all studies included an educational component. A meta-analysis of two studies determined that a multifaceted intervention (educational meetings and use of a mentor) did not increase engagement in a range of EIDM behaviours. Among the remaining studies, no definitive conclusions could be made about the relative effectiveness of the KT interventions due to variation of interventions and outcomes, as well as study limitations.
				**Target population:** nurses	**Scope of impact:** KT interventions are being implemented and evaluated on nurses’ behaviour and client outcomes. Implementing single-component educational interventions and multifaceted interventions with an educational component appear to have value for promoting nurses’ EIDM behaviours, while multifaceted interventions with an educational component were shown to contribute to improvements in client outcomes.
					**Quality of individual studies:** Most studies reporting quantitative data were at high risk of bias. Criteria judged across studies to be high risk of bias were primarily blinding of participants/personnel and other bias. Overall, the quality of the evidence ranged from very low to high. Most studies reporting qualitative information met the quality criteria.
**Multi-faceted Interventions (MFIs)**	**Albreacht L. et al.** *Systematic review of knowledge translation strategies to promote research uptake in child health settings* (2016)	7	21	**Setting:** HIC	**Effectiveness:** More than half of the included studies displayed mixed effects on primary outcome measures. Of the studies with moderate to strong methodological quality ratings, three demonstrated consistent, positive effect(s) on the primary outcome(s); effective knowledge translation interventions were two single, non-educational interventions and one multiple, educational intervention.
				**Target population:** health professional groups and settings	**Scope of impact:** Of the 21 included studies, the primary outcomes were professional/process outcomes (n = 14), patient outcomes (n = 1), and economic outcomes (n = 2). One study identified both professional/process and patient outcomes as primary outcomes and three studies did not clearly identify the primary outcome from multiple outcomes identified and measured.
					**Quality of individual studies:** The methodological quality of the included studies was largely moderate (n = 8) or weak (n = 11). Both studies rated as strong were RCTs. Six RCTs and two controlled before-after studies (CBAs) were rated as moderate. Five RCTs, two controlled clinical trials (CCTs), and four CBAs were classified as weak.
**Multi-faceted Interventions (MFIs)**	**Scott D. et al.** *Systematic review of knowledge translation strategies in the allied health professions* (2012)	7	32	**Setting:** HIC	**Effectiveness:** Education was the main approach for KT interventions. In the majority of studies, the interventions demonstrated mixed effects on primary outcomes, and only four studies demonstrated statistically significant, positive effects on primary outcomes.
				**Target population:** allied health professions	**Scope of impact:** The majority of primary outcomes were identified as professional/process outcomes (n = 25), but impact was limited. Patient outcomes (n = 4), economic outcomes (n = 2), and multiple primary outcomes (n = 1) were also represented.
					**Quality of individual studies:** Generally, the studies were of low methodological quality.
**Multi-faceted Interventions (MFIs)**	**Campbell A. et al.** *Knowledge translation strategies used by healthcare professionals in child health settings: an updated systematic review* (2019)	7	48	**Setting:** HIC	**Effectiveness:** Effective KT strategies used by health care professionals in child health settings were found to be online education curriculums and computerized decision supports or reminders. Findings of the review in conjunction with other knowledge syntheses examining multi versus single component KT interventions indicate that single KT interventions may be as effective or more effective than multicomponent interventions.
				**Target population:** health care professionals	**Scope of impact:** The most common primary outcomes were healthcare professional/process outcomes (n=32). Seven studies reported patient outcomes as the primary outcome and one study reported economic. Four studies reported healthcare professional/process and patient outcomes both as primary outcomes, one study reported healthcare professional/process, patient and economic outcomes as primary outcomes and three study's primary outcomes were unclear.
					**Quality of individual studies:** The methodological quality of studies was moderate (n=18), strong (n=16) and weak(n=14).
**Multi-faceted Interventions (MFIs)**	**Bird M.L. et al.** *Moving stroke rehabilitation evidence into practice: a systematic review of randomized controlled trials* (2019)	7	17	**Setting:** unclear/not specified	**Effectiveness:** Multicomponent multidisciplinary interventions that include site facilitation and consideration of local settings can change clinical practice. Also, education and training interventions should form part of multi-component interventions and not be used in isolation. Education is commonly used, but in isolation is not effective. Multicomponent interventions including facilitation and tailoring to local settings can change clinical practice and are more effective when targeting fewer changes.
				**Target population:** clinicians	**Scope of impact:** Implementing a small number of practice changes at a time produces more effective results.
					**Quality of individual studies:** Risk of bias was generally low. Overall, the GRADE (Grading of Recommendations assessment, Development and Evaluation) criteria indicated that this body of literature was of low quality.
**Multi-faceted Interventions (MFIs)**	**Goorts K., Dizon J. & Milanese S.** *The effectiveness of implementation strategies for promoting evidence informed interventions in allied healthcare: a systematic review* (2021)	7	6	**Setting:** HIC	**Effectiveness:** The review found moderate evidence for educational meetings, local opinion leaders and patient mediated interventions. The authors found stronger evidence for multifaceted components strategies. Few studies describe the effectiveness of implementation strategies for allied healthcare, but evidence was found for multi-faceted components for implementing research in an allied health therapy group population. When considering implementation of evidence informed interventions in allied health a multi-pronged approach appears to be more successful.
				**Target population:** allied health therapy group	**Scope of impact:** Multi-faceted strategies appear to remain the most effective in improving knowledge and adherence to guidelines and evidence (professional outcomes) but none of the strategies were found to improve patient outcomes.
					**Quality of individual studies:** The six studies included in this review were of sound methodologic quality with SIGN (Scottish Intercollegiate Guidelines Network) scores ranging from adequate to high quality.
**Multi-faceted Interventions (MFIs)**	**Zadro J.R. et al.** *Effectiveness of implementation strategies to improve adherence of physical therapist treatment choices to clinical practice guidelines for musculoskeletal conditions: systematic review* (2020)	7	9	**Setting:** HIC	**Effectiveness:** Four studies (out of 6 examining our primary outcome) identified implementation strategies that can increase physical therapists’ use of some evidence-based treatments. Although this review revealed limited trials evaluating interventions to increase physical therapists’ use of evidence-based treatments for musculoskeletal conditions compared with no intervention, it highlighted some interventions that may be effective.
				**Target population:** physical therapists	**Scope of impact:** There are examples where implementation strategies improved physical therapists’ use of evidence-based treatments despite most of the sample treating according to guidelines at baseline and where implementation strategies did not improve physical therapists’ use of evidence-based treatments despite most of the sample not following guidelines at baseline.
					**Quality of individual studies:** From the 9 studies, 2 studies were at low risk of bias for all EPOC domains. For random sequence generation, 2 were at unclear risk of bias and 7 were at low risk. For allocation concealment, 1 was at unclear risk of bias and 8 were at low risk. All studies were at low risk of bias for “baseline characteristics similar” and “selective outcome reporting,” whereas only 2 studies were at high risk of bias, and 1 had an unclear risk for baseline outcome measurements similar.” Six studies were at high risk of bias for “incomplete outcome data,” whereas 3 were at low risk. Eight studies ensured that knowledge of the allocated interventions were adequately prevented during the study (i.e., were at low risk of bias); 1 study was at high risk. Eight studies were at low risk of bias for protection against contamination, whereas only 1 was at high risk. One study assessed treatment choices using audits of clinical notes and was at low risk of “other bias”; the remaining 8 studies were at high risk of “other bias”.
**Multi-faceted Interventions (MFIs)**	**Menon A. et al.** *Strategies for rehabilitation professionals to move evidence-based knowledge into practice: a systematic review* (2009)	7	12	**Setting:** unclear/not specified	**Effectiveness:** Findings from this systematic review suggest that participation in an active multi-component KT intervention results in improved self-perceived knowledge, as well as positive changes in actual and self-perceived practice behaviors of physical therapists.
				**Target population:** rehabilitation clinicians	**Scope of impact:** These gains did not translate into change in physical therapists’ attitude towards best practices. While this review found no studies examining the use of active multi-component interventions with occupational therapists specifically, limited evidence suggests that single active KT interventions may improve knowledge, attitudes and practice behaviors of this professional group. It is not clear from this review which KT strategy can effectively change clinicians’ attitudes towards the use of EBP.
					**Quality of individual studies:** The most important limitation of this systematic review was the reported quality of the studies included, some of which had methodological weaknesses that may have reduced the validity of our conclusions for each PICO (population, intervention, control, outcomes) question.
**Multi-faceted Interventions (MFIs)**	**Jones C.A. et al.** *Translating knowledge in rehabilitation: systematic review* (2015)	7	26	**Setting:** HIC	**Effectiveness:** This systematic review showed that educational meetings were commonly used KT strategies specifically directed at translating research into practice and enhancing research uptake in rehabilitation disciplines. Active multicomponent KT strategies were effective in physical therapy knowledge and practice behaviors. Overall, the effectiveness of the KT strategies was not significant.
				**Target population:** rehabilitation professions	**Scope of impact:** No clear delineation of the effect on KT strategies was seen.
					**Quality of individual studies:** Based on the Quality Assessment Tool for Quantitative Studies, none of the quantitative studies received a strong rating, although 3 studies received a moderate rating and the remaining studies received weak ratings. The methodological rigor of most studies was weak.
**Multi-faceted Interventions (MFIs)**	**Van Der Veer S.N. et al.** *Translating knowledge on best practice into improving quality of RRT care: A systematic review of implementation strategies* (2011)	6	96	**Setting:** HIC	**Effectiveness:** Results tend to support previous findings that multifaceted strategies are more effective than single strategies.
				**Target population:** renal replacement therapy (RRT) providers	**Scope of impact:** The authors’ ability to draw firm conclusions on the relationship between the implementation strategies and specific QI techniques used and the outcomes achieved was limited.
					**Quality of individual studies:** Only a small number of studies were of sufficient methodological quality.
		**TOTAL**	**747**		

**Source:** Authors

**Table 2:** Strategy types used in included systematic reviews

Strategy type	Number	Authors
Educational strategies	3	Grudniewicz et al. [22]; Koota, Kääriäinen & Melender [23]; Wu et al. [20]
Local opinion leaders	1	Flodgren et al. [24]
Reminders	2	Arditi et al. [25]; Pantoja et al. [26]
ICT focused approaches	3	De Angelis et al. [27]; Brown et al. [28]; Jamal, McKenzie and Clark [14]
Audit and feedback	1	Sykes, McAnuff and Kolehmainen [29]
Non-EPOC listed strategies: Social media, Toolkits	2	Bhatt et al. [30]; Yamada et al. [31]
Multi-faceted interventions (MFIs)	20	Afari-Asiedu et al. [32]; Boonacker et al. [33]; Al Zoubi et al. [34]; Ariyo et al. [35]; Borgert, Goossens and Dongelmans [36]; Cahill et al. [37]; Pedersen et al. [38]; Jenkins et al. [39]; Bennett et al. [40]; Noonan et al. [41]; Yost et al. [21]; Albreacht et al. [42]; Scott et al. [19]; Campbell et al. [43]; Bird et al. [44]; Goorts, Dizon and Milanese [45]; Zadro et al. [46]; Menon et al. [15]; Jones et al. [18]; Van Der Veer et al. [47]

Source: authors

### Educational strategies

The overview identified three systematic reviews focusing on educational strategies. Grudniewicz et al. [22] explored the effectiveness of printed educational materials on primary care physician knowledge, behaviour and patient outcomes and concluded they were not effective in any of these aspects. Koota, Kääriäinen and Melender [23] focused on educational interventions promoting evidence-based practice among emergency room/accident and emergency nurses and found that interventions involving face-to-face contact led to significant or highly significant effects on patient benefits and emergency nurses’ knowledge, skills and behaviour. Interventions using written self-directed learning materials also led to significant improvements in nurses’ knowledge of evidence-based practice. Although the quality of the studies was high, the review primarily included small studies with low response rates, and many of them relied on self-assessed outcomes; consequently, the strength of the evidence for these outcomes is modest. Wu et al. [20] questioned if educational interventions aimed at nurses to support the implementation of evidence-based practice improve patient outcomes. Although based on evaluation projects and qualitative data, their results also suggest that positive changes on patient outcomes can be made following the implementation of specific evidence-based approaches (or projects). The differing positive outcomes for educational strategies aimed at nurses might indicate that the target audience is important.

### Local opinion leaders

Flodgren et al. [24] was the only systemic review focusing solely on opinion leaders. The review found that local opinion leaders alone, or in combination with other interventions, can be effective in promoting evidence‐based practice, but this varies both within and between studies and the effect on patient outcomes is uncertain. The review found that, overall, any intervention involving opinion leaders probably improves healthcare professionals’ compliance with evidence-based practice but varies within and across studies. However, how opinion leaders had an impact could not be determined because of insufficient details were provided, illustrating that reporting specific details in published studies is important if diffusion of effective methods of increasing evidence-based practice is to be spread across a system. The usefulness of this review is questionable because it cannot provide evidence of what is an effective opinion leader, whether teams of opinion leaders or a single opinion leader are most effective, or the most effective methods used by opinion leaders.

### Reminders

Pantoja et al. [26] was the only systemic review focusing solely on manually generated reminders delivered on paper included in the overview. The review explored how these affected professional practice and patient outcomes. The review concluded that manually generated reminders delivered on paper as a single intervention probably led to small to moderate increases in adherence to clinical recommendations, and they could be used as a single quality improvement intervention. However, the authors indicated that this intervention would make little or no difference to patient outcomes. The authors state that such a low-tech intervention may be useful in low- and middle-income countries where paper records are more likely to be the norm.

### ICT-focused approaches

The three ICT-focused reviews [14, 27, 28] showed mixed results. Jamal, McKenzie and Clark [14] explored the impact of health information technology on the quality of medical and health care. They examined the impact of electronic health record, computerised provider order-entry, or decision support system. This showed a positive improvement in adherence to evidence-based guidelines but not to patient outcomes. The number of studies included in the review was low and so a conclusive recommendation could not be reached based on this review. Similarly, Brown et al. [28] found that technology-enabled knowledge translation interventions may improve knowledge of health professionals, but all eight studies raised concerns of bias. The De Angelis et al. [27] review was more promising, reporting that ICT can be a good way of disseminating clinical practice guidelines but conclude that it is unclear which type of ICT method is the most effective.

### Audit and feedback

Sykes, McAnuff and Kolehmainen [29] examined whether audit and feedback were effective in dementia care and concluded that it remains unclear which ingredients of audit and feedback are successful as the reviewed papers illustrated large variations in the effectiveness of interventions using audit and feedback.

### Non-EPOC listed strategies: social media, toolkits

There were two new (non-EPOC listed) intervention types identified in this review compared to the 2011 review — fewer than anticipated. We categorised a third — ‘care bundles’ [36] as a multi-faceted intervention due to its description in practice and a fourth — ‘Technology Enhanced Knowledge Transfer’ [28] was classified as an ICT-focused approach. The first new strategy was identified in Bhatt et al.’s [30] systematic review of the use of social media for the dissemination of clinical practice guidelines. They reported that the use of social media resulted in a significant improvement in knowledge and compliance with evidence-based guidelines compared with more traditional methods. They noted that a wide selection of different healthcare professionals and patients engaged with this type of social media and its global reach may be significant for low- and middle-income countries. This review was also noteworthy for developing a simple stepwise method for using social media for the dissemination of clinical practice guidelines. However, it is debatable whether social media can be classified as an intervention or just a different way of delivering an intervention. For example, the review discussed involving opinion leaders and patient advocates through social media. However, this was a small review that included only five studies, so further research in this new area is needed. Yamada et al. [31] draw on 39 studies to explore the application of toolkits, 18 of which had toolkits embedded within larger KT interventions, and 21 of which evaluated toolkits as standalone interventions. The individual component strategies of the toolkits were highly variable though the authors suggest that they align most closely with educational strategies. The authors conclude that toolkits as either standalone strategies or as part of MFIs hold some promise for facilitating evidence use in practice but caution that the quality of many of the primary studies included is considered weak limiting these findings.

### Multi-faceted interventions

The majority of the systematic reviews (*n* = 20) reported on more than one intervention type. Some of these systematic reviews focus exclusively on multi-faceted interventions, whilst others compare different single or combined interventions aimed at achieving similar outcomes in particular settings. While these two approaches are often described in a similar way, they are actually quite distinct from each other as the former report how multiple strategies may be strategically combined in pursuance of an agreed goal, whilst the latter report how different strategies may be incidentally used in sometimes contrasting settings in the pursuance of similar goals. Ariyo et al. [35] helpfully summarise five key elements often found in effective MFI strategies in LMICs — but which may also be transferrable to HICs. First, effective MFIs encourage a multi-disciplinary approach acknowledging the roles played by different professional groups to collectively incorporate evidence-informed practice. Second, they utilise leadership drawing on a wide set of clinical and non-clinical actors including managers and even government officials. Third, multiple types of educational practices are utilised — including input from patients as stakeholders in some cases. Fourth, protocols, checklists and bundles are used — most effectively when local ownership is encouraged. Finally, most MFIs included an emphasis on monitoring and evaluation [35]. In contrast, other studies offer little information about the nature of the different MFI components of included studies which makes it difficult to extrapolate much learning from them in relation to why or how MFIs might affect practice (e.g. [28, 38]). Ultimately, context matters, which some review authors argue makes it difficult to say with real certainty whether single or MFI strategies are superior (e.g. [21, 27]). Taking all the systematic reviews together we may conclude that MFIs appear to be more likely to generate positive results than single interventions (e.g. [34, 45]) though other reviews should make us cautious (e.g. [32, 43]).

## Discussion

While multi-faceted interventions still seem to be more effective than single-strategy interventions, there were important distinctions between how the results of reviews of MFIs are interpreted in this review as compared to the previous reviews [8, 9], reflecting greater nuance and debate in the literature. This was particularly noticeable where the effectiveness of MFIs was compared to single strategies, reflecting developments widely discussed in previous studies [10]. We found that most systematic reviews are bounded by their clinical, professional, spatial, system, or setting criteria and often seek to draw out implications for the implementation of evidence in their areas of specific interest (such as nursing or acute care). Frequently this means combining all relevant studies to explore the respective foci of each systematic review. Therefore, most reviews we categorised as MFIs actually include highly variable numbers and combinations of intervention strategies and highly heterogeneous original study designs. This makes statistical analyses of the type used by Squires et al. [10] on the three reviews in their paper not possible. Further, it also makes extrapolating findings and commenting on broad themes complex and difficult. This may suggest that future research should shift its focus from merely examining ‘what works’ to ‘what works where and what works for whom’ — perhaps pointing to the value of realist approaches to these complex review topics [48, 49] and other more theory-informed approaches [50].

Some reviews have a relatively small number of studies (i.e. fewer than 10) and the authors are often understandably reluctant to engage with wider debates about the implications of their findings. Other larger studies do engage in deeper discussions about internal comparisons of findings across included studies and also contextualise these in wider debates. Some of the most informative studies (e.g. [35, 40]) move beyond EPOC categories and contextualise MFIs within wider systems thinking and implementation theory. This distinction between MFIs and single interventions can actually be very useful as it offers lessons about the contexts in which individual interventions might have bounded effectiveness (i.e. educational interventions for individual change). Taken as a whole, this may also then help in terms of how and when to conjoin single interventions into effective MFIs.

In the two previous reviews, a consistent finding was that MFIs were more effective than single interventions [8, 9]. However, like Squires et al. [10] this overview is more equivocal on this important issue. There are four points which may help account for the differences in findings in this regard. Firstly, the diversity of the systematic reviews in terms of clinical topic or setting is an important factor. Secondly, there is heterogeneity of the studies within the included systematic reviews themselves. Thirdly, there is a lack of consistency with regards to the definition and strategies included within of MFIs. Finally, there are epistemological differences across the papers and the reviews. This means that the results that are presented depend on the methods used to measure, report, and synthesise them. For instance, some reviews highlight that education strategies can be useful to improve provider understanding — but without wider organisational or system-level change, they may struggle to deliver sustained transformation [19, 44].

It is also worth highlighting the importance of the theory of change underlying the different interventions. Where authors of the systematic reviews draw on theory, there is space to discuss/explain findings. We note a distinction between theoretical and atheoretical systematic review discussion sections. Atheoretical reviews tend to present acontextual findings (for instance, one study found very positive results for one intervention, and this gets highlighted in the abstract) whilst theoretically informed reviews attempt to contextualise and explain patterns within the included studies. Theory-informed systematic reviews seem more likely to offer more profound and useful insights (see [19, 35, 40, 43, 45]). We find that the most insightful systematic reviews of MFIs engage in theoretical generalisation — they attempt to go beyond the data of individual studies and discuss the wider implications of the findings of the studies within their reviews drawing on implementation theory. At the same time, they highlight the active role of context and the wider relational and system-wide issues linked to implementation. It is these types of investigations that can help providers further develop evidence-based practice.

This overview has identified a small, but insightful set of papers that interrogate and help theorise why, how, for whom, and in which circumstances it might be the case that MFIs are superior (see [19, 35, 40] once more). At the level of this overview — and in most of the systematic reviews included — it appears to be the case that MFIs struggle with the question of attribution. In addition, there are other important elements that are often unmeasured, or unreported (e.g. costs of the intervention — see [40]). Finally, the stronger systematic reviews [19, 35, 40, 43, 45] engage with systems issues, human agency and context [18] in a way that was not evident in the systematic reviews identified in the previous reviews [8, 9]. The earlier reviews lacked any theory of change that might explain why MFIs might be more effective than single ones — whereas now some systematic reviews do this, which enables them to conclude that sometimes single interventions can still be more effective.

As Nilsen et al. ([6] p. 7) note ‘Study findings concerning the effectiveness of various approaches are continuously synthesized and assembled in systematic reviews’. We may have gone as far as we can in understanding the implementation of evidence through systematic reviews of single and multi-faceted interventions and the next step would be to conduct more research exploring the complex and situated nature of evidence used in clinical practice and by particular professional groups. This would further build on the nuanced discussion and conclusion sections in a subset of the papers we reviewed. This might also support the field to move away from isolating individual implementation strategies [6] to explore the complex processes involving a range of actors with differing capacities [51] working in diverse organisational cultures. Taxonomies of implementation strategies do not fully account for the complex process of implementation, which involves a range of different actors with different capacities and skills across multiple system levels. There is plenty of work to build on, particularly in the social sciences, which currently sits at the margins of debates about evidence implementation (see for example, Normalisation Process Theory [52]).

There are several changes that we have identified in this overview of systematic reviews in comparison to the review we published in 2011 [8]. A consistent and welcome finding is that the overall quality of the systematic reviews themselves appears to have improved between the two reviews, although this is not reflected upon in the papers. This is exhibited through better, clearer reporting mechanisms in relation to the mechanics of the reviews, alongside a greater attention to, and deeper description of, how potential biases in included papers are discussed. Additionally, there is an increased, but still limited, inclusion of original studies conducted in low- and middle-income countries as opposed to just high-income countries. Importantly, we found that many of these systematic reviews are attuned to, and comment upon the contextual distinctions of pursuing evidence-informed interventions in health care settings in different economic settings. Furthermore, systematic reviews included in this updated article cover a wider set of clinical specialities (both within and beyond hospital settings) and have a focus on a wider set of healthcare professions — discussing both similarities, differences and inter-professional challenges faced therein, compared to the earlier reviews. These wider ranges of studies highlight that a particular intervention or group of interventions may work well for one professional group but be ineffective for another. This diversity of study settings allows us to consider the important role context (in its many forms) plays on implementing evidence into practice. Examining the complex and varied context of health care will help us address what Nilsen et al. ([6] p. 1) described as, ‘society’s health problems [that] require research-based knowledge acted on by healthcare practitioners together with implementation of political measures from governmental agencies’. This will help us shift implementation science to move, ‘beyond a success or failure perspective towards improved analysis of variables that could explain the impact of the implementation process’ ([6] p. 2).

## Conclusion

This review brings together 32 papers considering individual and multi-faceted interventions designed to support the use of evidence in clinical practice. The majority of reviews report strategies achieving small impacts (normally on processes of care). There is much less evidence that these strategies have shifted patient outcomes. Combined with the two previous reviews, 86 systematic reviews of strategies to increase the implementation of research into clinical practice have been conducted. As a whole, this substantial body of knowledge struggles to tell us more about the use of individual and MFIs than: ‘it depends’. To really move forwards in addressing the gap between research evidence and practice, we may need to shift the emphasis away from isolating individual and multi-faceted interventions to better understanding and building more situated, relational and organisational capability to support the use of research in clinical practice. This will involve drawing on a wider range of perspectives, especially from the social, economic, political and behavioural sciences in primary studies and diversifying the types of synthesis undertaken to include approaches such as realist synthesis which facilitate exploration of the context in which strategies are employed. Harvey et al. [53] suggest that when context is likely to be critical to implementation success there are a range of primary research approaches (participatory research, realist evaluation, developmental evaluation, ethnography, quality/ rapid cycle improvement) that are likely to be appropriate and insightful. While these approaches often form part of implementation studies in the form of process evaluations, they are usually relatively small scale in relation to implementation research as a whole. As a result, the findings often do not make it into the subsequent systematic reviews. This review provides further evidence that we need to bring qualitative approaches in from the periphery to play a central role in many implementation studies and subsequent evidence syntheses. It would be helpful for systematic reviews, at the very least, to include more detail about the interventions and their implementation in terms of how and why they worked.

### Supplementary Information

**Additional file 1: Appendix A.**

**Additional file 2: Appendix B.**

## Data Availability

The datasets used and/or analysed during the current study are available from the corresponding author on reasonable request.

## Abbreviations

BA: Before and after study
CCT: Controlled clinical trial
EPOC: Effective Practice and Organisation of Care
HICs: High-income countries
ICT: Information and Communications Technology
ITS: Interrupted time series
KT: Knowledge translation
LMICs: Low- and middle-income countries
MFIs: Multi-faceted interventions
RCT: Randomised controlled trial
