# Supplementary material for: ‘It depends’: what 86 systematic reviews tell us about what strategies to use to support the use of research in clinical practice
Source: Implement Sci. 2024 Feb 19;19:15. doi: 10.1186/s13012-024-01337-z (PMC10875780; doi:10.1186/s13012-024-01337-z)
Supplement: Supplementary file 1 — Additional file 1: Appendix A. [file 13012_2024_1337_MOESM1_ESM.docx]

# **Additional file 1**

# **Appendix A:** Search strategy

This appendix provides full details of all search strings used for bibliographic databases and trials registers, with dates and number of references returned and notes explaining any unusual search techniques or syntax. The EndNote 20 import order is provided, as the deduplication technique keeps the first uploaded copy of the reference by default. All results from Search 1 were uploaded to EndNote before the results from Search 2 were uploaded.

## OvidSP Medline ALL

| Database name | Medline ALL |
| --- | --- |
| Database platform | OvidSP |
| Dates of database coverage | 1946 to June 17, 2022 |
| Date searched | 2022-06-20 |
| Searched by | JF |
| Number of results | Search 1: 15,999 Search 2: 3219 |
| EndNote import order | 1 (Medline) 3 (Medline in-process and other items) |
| Number of results once duplicates removed | Search 1: 14,416 Search 2: 3064 |
| Search strategy notes | Search lines ending in a ‘/’ are subject heading searches. Search lines beginning ‘exp’ are exploded subject heading searches. Subject heading searched beginning with a ‘*’ are focused subject headings. Two-letter codes at the end of search lines designate the fields to search. Fields codes used are:  AB: abstract   TI: title  TW: textword or/*x-y* combines search sets in the range *x-y* with Boolean operator OR. $ or * is used for truncation of words. ? finds 0 or 1 character in that place. adj*n* finds words within *n* words of each other, in any order. Words in square brackets [ … ] are comments and are ignored by the database search algorithm. |

| # | Search terms | Results |
| --- | --- | --- |
| 1 | exp Education, Continuing/ | 62410 |
| 2 | (education$ adj2 (program$ or intervention? or meeting? or session? or strateg$ or workshop? or visit?)).tw. | 80184 |
| 3 | *Pamphlets/ | 1871 |
| 4 | (behavio?r$ adj2 intervention?).tw. | 18212 |
| 5 | (leaflet? or booklet? or poster or posters).tw. | 36351 |
| 6 | ((written or printed or oral) adj information).tw. | 2459 |
| 7 | (information$ adj2 campaign?).tw. | 1397 |
| 8 | (education$ adj1 (method? or material?)).tw. | 9272 |
| 9 | outreach.tw. | 16176 |
| 10 | ((opinion or education$ or influential) adj1 leader?).tw. | 2051 |
| 11 | facilitator?.tw. | 33295 |
| 12 | academic detailing.tw. | 598 |
| 13 | consensus conference?.tw. | 5965 |
| 14 | practice guideline?.tw. | 30042 |
| 15 | (guideline? adj2 (introduc$ or issu$ or impact or effect? or disseminat$ or distribut$)).tw. | 6073 |
| 16 | ((effect? or impact or evaluat$ or introduc$ or compar$) adj2 training program$).tw. | 1084 |
| 17 | *reminder systems/ | 2264 |
| 18 | reminder?.tw. | 14923 |
| 19 | (recall adj2 system$).tw. | 567 |
| 20 | (prompter? or prompting).tw. | 11069 |
| 21 | algorith?.tw. | 228148 |
| 22 | *feedback/ or feedback.tw. | 159934 |
| 23 | (feedback adj1 (loop? or control? or regula$ or mechanism? or inhib$ or system? or circuit? or sensory or visual or audi$)).tw. | 55623 |
| 24 | 22 not 23 | 104311 |
| 25 | chart review$.tw. | 49339 |
| 26 | ((effect? or impact or records or chart?) adj2 audit).tw. | 1236 |
| 27 | *patient education/ | 40825 |
| 28 | marketing.tw. | 29460 |
| 29 | ((effect? or impact or evaluat? or introduc$ or compara$) adj2 (prevent$ program$ or screening program$)).tw. | 699 |
| 30 | ((introduc$ or impact or effect? or implement$ or computer$) adj2 protocol?).tw. | 5879 |
| 31 | (computer$ adj2 (dosage or dosing or diagnosis or therapy or decision?)).tw. | 7176 |
| 32 | *physician's practice patterns/ | 41084 |
| 33 | or/1-21,24-32 | 787076 |
| 34 | ((systematic* and review) or systematic overview* or cochrane review* or systemic review* or scoping review or scoping literature review or mapping review or umbrella review* or "review of reviews" or "overview of reviews" or meta-review or integrative review or integrated review or integrative overview or meta-synthesis or metasynthesis or quantitative review or quantitative synthesis or research synthesis or meta-ethnography or systematic literature search or systematic literature research or meta-analyses or metaanalyses or metaanalysis or meta-analysis or meta-analytic review or meta-analytical review).ti. | 287858 |
| 35 | (meta-analysis or systematic review).pt. | 275820 |
| 36 | ((search* or medline or pubmed or embase or cochrane or scopus or "web of science" or "sources of information" or data sources or following databases) and (study selection or selection criteria or eligibility criteria or inclusion criteria or exclusion criteria)).ti,ab. | 100682 |
| 37 | or/34-36 | 374357 |
| 38 | (letter or editorial or comment or case reports or historical article or "retraction of publication" or retracted publication or published erratum).pt. or (reply or report or protocol or protocols or withdrawn).ti. or "retraction of publication as topic"/ | 4904576 |
| 39 | 37 not 38 | 346316 |
| 40 | 33 and 39 | 18091 |
| 41 | exp animals/ not humans.sh. | 5019208 |
| 42 | 40 not 41 | 18053 |
| 43 | limit 42 to yr="2009 -Current" [Search 1 results] | 15999 |
| 44 | ((digital* or non-digital* or cyber* or computer* or computing) adj2 (introduc$ or impact or effect? or evaluat$ or implement$ or decision?)).ti,ab,kf. | 14503 |
| 45 | ((((information or communicat*) adj technolog*) or ICT) adj2 (introduc$ or impact or effect? or evaluat$ or implement$ or decision?)).ti,ab,kf. | 1087 |
| 46 | diffusion of innovation/ or technology transfer/ | 20195 |
| 47 | (technolo* adj1 (advance* or develop* or new or innovat* or emerging or transfer* or licens*) adj2 (introduc$ or impact or effect? or evaluat$ or implement$ or decision?)).ti,ab,kf. | 2781 |
| 48 | exp *cell phone/ | 14438 |
| 49 | ((((mobile or cell or cellular) adj1 (phone* or telephone* or handset* or device*)) or text messag* or SMS or texting) adj2 (introduc$ or impact or effect? or evaluat$ or implement$ or decision?)).ti,ab,kf. | 1516 |
| 50 | exp *computers, handheld/ | 7350 |
| 51 | ((smartphone or smart phone or ((handheld or hand-held or mobile) adj1 device*) or mobile handset*) adj2 (introduc$ or impact or effect? or evaluat$ or implement$ or decision?)).ti,ab,kf. | 641 |
| 52 | exp *software/ | 83752 |
| 53 | (software adj2 (introduc$ or impact or effect? or evaluat$ or implement$ or decision?)).ti,ab,kf. | 5003 |
| 54 | *data mining/ | 5634 |
| 55 | ((((data or text) adj1 mining) or data dashboard*) adj2 (introduc$ or impact or effect? or evaluat$ or implement$ or decision?)).ti,ab,kf. | 228 |
| 56 | *big data/ | 1279 |
| 57 | exp *Algorithms/ | 169521 |
| 58 | ((algorithms or artificial intelligence or (machine* adj1 (intelligen* or learn*)) or AI or expert systems or fuzzy logic or deep learning or robotics or natural language processing) adj2 (introduc$ or impact or effect? or evaluat$ or implement$ or decision?)).ti,ab,kf. | 9092 |
| 59 | *Electronic data processing/ | 7217 |
| 60 | ((((electronic or automatic) adj2 (data or information)) or optical reader* or bar code* or qr code*) adj2 (introduc$ or impact or effect? or evaluat$ or implement$ or decision?)).ti,ab,kf. | 243 |
| 61 | (cloud adj1 (comput* or process* or service* or stor*) adj2 (introduc$ or impact or effect? or evaluat$ or implement$ or decision?)).ti,ab,kf. | 24 |
| 62 | or/44-61 | 316086 |
| 63 | 39 and 62 | 4423 |
| 64 | 63 not 41 | 4403 |
| 65 | limit 64 to yr="2009 -Current" | 4108 |
| 66 | 65 not 43 [Search 2 results] | 3219 |

## OvidSP Embase

| Database name | Embase |
| --- | --- |
| Database platform | OvidSP |
| Dates of database coverage | 1980 to 2022 week 24 |
| Date searched | 2022-06-20 |
| Searched by | JF |
| Number of results | Search 1: 26,797 Search 2: 4977 |
| EndNote import order | 1 (Medline) 3 (Medline in-process and other items) |
| Number of results once duplicates removed | Search 1: 14,258 Search 2: 915 |
| Search strategy notes | Search lines ending in a ‘/’ are subject heading searches. Search lines beginning ‘exp’ are exploded subject heading searches. Subject heading searched beginning with a ‘*’ are focused subject headings. Two-letter codes at the end of search lines designate the fields to search. Fields codes used are:  AB: abstract   TI: title  TW: textword or/*x-y* combines search sets in the range *x-y* with Boolean operator OR. $ or * is used for truncation of words. ? finds 0 or 1 character in that place. adj*n* finds words within *n* words of each other, in any order. Words in square brackets [ … ] are comments and are ignored by the database search algorithm. |

| # | Search terms | Results |
| --- | --- | --- |
| 1 | continuing education/ | 31802 |
| 2 | (education$ adj2 (program$ or intervention? or meeting? or session? or strateg$ or workshop? or visit?)).tw. | 109286 |
| 3 | *publication/ | 41976 |
| 4 | (behavio?r$ adj2 intervention?).tw. | 23041 |
| 5 | (leaflet? or booklet? or poster or posters).tw. | 62769 |
| 6 | ((written or printed or oral) adj information).tw. | 4218 |
| 7 | (information$ adj2 campaign?).tw. | 1765 |
| 8 | (education$ adj1 (method? or material?)).tw. | 16932 |
| 9 | outreach.tw. | 22924 |
| 10 | ((opinion or education$ or influential) adj1 leader?).tw. | 2714 |
| 11 | facilitator?.tw. | 40704 |
| 12 | academic detailing.tw. | 861 |
| 13 | consensus conference?.tw. | 8235 |
| 14 | practice guideline?.tw. | 41860 |
| 15 | (guideline? adj2 (introduc$ or issu$ or impact or effect? or disseminat$ or distribut$)).tw. | 10640 |
| 16 | ((effect? or impact or evaluat$ or introduc$ or compar$) adj2 training program$).tw. | 1431 |
| 17 | *reminder system/ | 1240 |
| 18 | reminder?.tw. | 22787 |
| 19 | (recall adj2 system$).tw. | 691 |
| 20 | (prompter? or prompting).tw. | 17142 |
| 21 | algorith?.tw. | 290495 |
| 22 | exp *feedback system/ | 22677 |
| 23 | feedback.tw. | 200387 |
| 24 | (feedback adj1 (loop? or control? or regula$ or mechanism? or inhib$ or system? or circuit? or sensory or visual or audi$)).tw. | 62957 |
| 25 | (22 or 23) not 24 | 143890 |
| 26 | chart review$.tw. | 102991 |
| 27 | ((effect? or impact or records or chart?) adj2 audit).tw. | 2111 |
| 28 | *patient education/ | 29921 |
| 29 | marketing.tw. | 40424 |
| 30 | ((effect? or impact or evaluat? or introduc$ or compara$) adj2 (prevent$ program$ or screening program$)).tw. | 949 |
| 31 | ((introduc$ or impact or effect? or implement$ or computer$) adj2 protocol?).tw. | 9419 |
| 32 | (computer$ adj2 (dosage or dosing or diagnosis or therapy or decision?)).tw. | 8751 |
| 33 | *clinical practice/ | 48357 |
| 34 | or/1-21,25-33 | 1067067 |
| 35 | ((systematic* and review) or systematic overview* or cochrane review* or systemic review* or scoping review or scoping literature review or mapping review or umbrella review* or "review of reviews" or "overview of reviews" or meta-review or integrative review or integrated review or integrative overview or meta-synthesis or metasynthesis or quantitative review or quantitative synthesis or research synthesis or meta-ethnography or systematic literature search or systematic literature research or meta-analyses or metaanalyses or metaanalysis or meta-analysis or meta-analytic review or meta-analytical review).ti. | 344189 |
| 36 | "systematic review"/ | 348544 |
| 37 | exp meta analysis/ | 248768 |
| 38 | ((search* or medline or pubmed or embase or cochrane or scopus or "web of science" or "sources of information" or data sources or following databases) and (study selection or selection criteria or eligibility criteria or inclusion criteria or exclusion criteria)).ti,ab. | 122532 |
| 39 | (editorial or erratum or letter or tombstone).pt. or (reply or report or protocol or protocols or withdrawn).ti. or exp erratum/ | 2821760 |
| 40 | or/35-38 | 562622 |
| 41 | 40 not 39 | 529514 |
| 42 | 34 and 41 | 29706 |
| 43 | (rat or rats or mouse or mice or swine or porcine or murine or sheep or lambs or pigs or piglets or rabbit or rabbits or cat or cats or dog or dogs or cattle or bovine or monkey or monkeys or trout or marmoset$1).ti. and animal experiment/ | 1105899 |
| 44 | Animal experiment/ not (human experiment/ or human/) | 2293710 |
| 45 | 43 or 44 | 2353320 |
| 46 | 42 not 45 | 29697 |
| 47 | limit 46 to yr="2009 -Current" [Search 1 results] | 26797 |
| 48 | ((digital* or non-digital* or cyber* or computer* or computing) adj2 (introduc$ or impact or effect? or evaluat$ or implement$ or decision?)).ti,ab,kf. | 17228 |
| 49 | ((((information or communicat*) adj technolog*) or ICT) adj2 (introduc$ or impact or effect? or evaluat$ or implement$ or decision?)).ti,ab,kf. | 1255 |
| 50 | "diffusion of innovation"/ | 63 |
| 51 | (technolo* adj1 (advance* or develop* or new or innovat* or emerging or transfer* or licens*) adj2 (introduc$ or impact or effect? or evaluat$ or implement$ or decision?)).ti,ab,kf. | 3865 |
| 52 | exp *mobile phone/ | 15287 |
| 53 | ((((mobile or cell or cellular) adj1 (phone* or telephone* or handset* or device*)) or text messag* or SMS or texting) adj2 (introduc$ or impact or effect? or evaluat$ or implement$ or decision?)).ti,ab,kf. | 1878 |
| 54 | *personal digital assistant/ or *tablet computer/ | 1749 |
| 55 | ((smartphone or smart phone or ((handheld or hand-held or mobile) adj1 device*) or mobile handset*) adj2 (introduc$ or impact or effect? or evaluat$ or implement$ or decision?)).ti,ab,kf. | 835 |
| 56 | exp *software/ | 33720 |
| 57 | (software adj2 (introduc$ or impact or effect? or evaluat$ or implement$ or decision?)).ti,ab,kf. | 6793 |
| 58 | *data mining/ | 5118 |
| 59 | ((((data or text) adj1 mining) or data dashboard*) adj2 (introduc$ or impact or effect? or evaluat$ or implement$ or decision?)).ti,ab,kf. | 277 |
| 60 | *machine learning/ | 26318 |
| 61 | *big data/ | 2106 |
| 62 | exp *algorithm/ | 136432 |
| 63 | ((algorithms or artificial intelligence or (machine* adj1 (intelligen* or learn*)) or AI or expert systems or fuzzy logic or deep learning or robotics or natural language processing) adj2 (introduc$ or impact or effect? or evaluat$ or implement$ or decision?)).ti,ab,kf. | 11110 |
| 64 | *cloud computing/ or *computer analysis/ | 5652 |
| 65 | ((((electronic or automatic) adj2 (data or information)) or optical reader* or bar code* or qr code*) adj2 (introduc$ or impact or effect? or evaluat$ or implement$ or decision?)).ti,ab,kf. | 340 |
| 66 | (cloud adj1 (comput* or process* or service* or stor*) adj2 (introduc$ or impact or effect? or evaluat$ or implement$ or decision?)).ti,ab,kf. | 49 |
| 67 | or/48-66 | 252545 |
| 68 | 41 and 67 | 5131 |
| 69 | 68 not 45 | 5130 |
| 70 | limit 69 to yr="2009 -Current" [Search 2 results] | 4977 |

## Wiley Cochrane Database of Systematic Reviews

| Database name | Cochrane Databases of Systematic Reviews (CDSR) |
| --- | --- |
| Database platform | Wiley Cochrane Library |
| Dates of database coverage | Issue 6 of 12, June 2022 |
| Date searched | 2022-06-20 |
| Searched by | JF |
| Number of results | Search 1: 795 Search 2: 41  Note, number of results below are for all Cochrane Library sections. |
| EndNote import order | 4 |
| Number of results once duplicates removed | Search 1: 206 Search 2: 15 |
| Search strategy notes | Search lines beginning ‘MeSH descriptor’ are subject heading searches. Search lines ending ‘explode all trees’ are exploded subject heading searches. Search lines ending ‘this term only’ are not exploded subject heading searches. Search lines ending ‘:ti,ab,kw’ search the title, abstract and keywords. {OR *#x-#y*} combines search sets in the range *x-y* with Boolean operator OR. * is used for truncation of words. ? finds 0 or 1 character in that place. NEAR/*n* finds words within *n* words of each other, in any order. NEXT finds words next to each other, in the order entered. |

| # | Search terms | Results |
| --- | --- | --- |
| #1 | MeSH descriptor: [Education, Continuing] explode all trees | 1180 |
| #2 | (education* NEAR/2 (program* or intervention? or meeting? or session? or strateg* or workshop? or visit?)):ti,ab,kw | 21926 |
| #3 | MeSH descriptor: [Pamphlets] this term only | 934 |
| #4 | (behavio?r* NEAR/2 intervention?):ti,ab,kw | 11118 |
| #5 | (leaflet? or booklet? or poster or posters):ti,ab,kw | 7445 |
| #6 | ((written or printed or oral) NEAR/1 information):ti,ab,kw | 1603 |
| #7 | (information* NEAR/2 campaign?):ti,ab,kw | 81 |
| #8 | (education* NEAR/1 (method? or material?)):ti,ab,kw | 6110 |
| #9 | (outreach):ti,ab,kw | 2314 |
| #10 | ((opinion or education* or influential) NEAR/1 leader?):ti,ab,kw | 265 |
| #11 | (facilitator?):ti,ab,kw | 4066 |
| #12 | ("academic detailing"):ti,ab,kw | 311 |
| #13 | (consensus NEXT conference?):ti,ab,kw | 347 |
| #14 | (practice NEXT guideline?):ti,ab,kw | 14008 |
| #15 | (guideline? NEAR/2 (introduc* or issu* or impact or effect? or disseminat* or distribut*)):ti,ab,kw | 870 |
| #16 | ((effect? or impact or evaluat* or introduc* or compar*) NEAR/2 (training NEXT program*)):ti,ab,kw | 381 |
| #17 | MeSH descriptor: [Reminder Systems] this term only | 1016 |
| #18 | (reminder?):ti,ab,kw | 6358 |
| #19 | (recall NEAR/2 system*):ti,ab,kw | 121 |
| #20 | (prompter? or prompting):ti,ab,kw | 1000 |
| #21 | (algorith?):ti,ab,kw | 11319 |
| #22 | MeSH descriptor: [Feedback] this term only | 1464 |
| #23 | (feedback):ti,ab,kw | 19957 |
| #24 | (feedback NEAR/1 (loop? or control? or regula* or mechanism? or inhib* or system? or circuit? or sensory or visual or audi*)):ti,ab,kw | 4773 |
| #25 | (#22 OR #23) NOT #24 | 15184 |
| #26 | (chart NEXT review*):ti,ab,kw | 2299 |
| #27 | ((effect? or impact or records or chart?) NEAR/2 audit):ti,ab,kw | 189 |
| #28 | MeSH descriptor: [Patient Education as Topic] this term only | 9229 |
| #29 | (marketing):ti,ab,kw | 2541 |
| #30 | ((effect? or impact or evaluat? or introduc* or compara*) NEAR/2 ((prevent* NEXT program*) or (screening NEXT program*))):ti,ab,kw | 109 |
| #31 | ((introduc* or impact or effect? or implement* or computer*) NEAR/2 protocol?):ti,ab,kw | 5868 |
| #32 | (computer* NEAR/2 (dosage or dosing or diagnosis or therapy or decision?)):ti,ab,kw | 3901 |
| #33 | MeSH descriptor: [Practice Patterns, Physicians'] this term only | 1307 |
| #34 | #1 OR #2 OR #3 OR #4 OR #5 OR #6 OR #7 OR #8 OR #9 OR #10 OR #11 OR #12 OR #13 OR #14 OR #15 OR #16 OR #17 OR #18 OR #19 OR #20 OR #21 OR #25 OR #26 OR #27 OR #28 OR #29 OR #30 OR #31 OR #32 OR #33 | 109634 |
| #35 | ((digital* or non-digital* or cyber* or computer* or computing) near/2 (introduc* or impact or effect? or evaluat* or implement* or decision?)):ti,ab,kw | 2564 |
| #36 | ((((information or communicat*) near/1 technolog*) or ICT) NEAR/2 (introduc* or impact or effect? or evaluat* or implement* or decision?)):ti,ab,kw | 83 |
| #37 | MeSH descriptor: [Diffusion of Innovation] this term only | 136 |
| #38 | MeSH descriptor: [Technology Transfer] this term only | 12 |
| #39 | (technolo* NEAR/1 (advance* or develop* or new or innovat* or emerging or transfer* or licens*) NEAR/2 (introduc* or impact or effect? or evaluat* or implement* or decision?)):ti,ab,kw | 126 |
| #40 | MeSH descriptor: [Cell Phone] explode all trees | 2265 |
| #41 | ((((mobile or cell or cellular) NEAR/1 (phone* or telephone* or handset* or device*)) or (text NEXT messag*) or SMS or texting) NEAR/2 (introduc* or impact or effect? or evaluat* or implement* or decision?)):ti,ab,kw | 498 |
| #42 | MeSH descriptor: [Computers, Handheld] explode all trees | 956 |
| #43 | ((smartphone or "smart phone" or ((handheld or hand-held or mobile) NEAR/1 device*) or (mobile NEXT handset*)) NEAR/2 (introduc* or impact or effect? or evaluat* or implement* or decision?)):ti,ab,kw | 282 |
| #44 | MeSH descriptor: [Software] explode all trees | 4125 |
| #45 | (software NEAR/2 (introduc* or impact or effect? or evaluat* or implement* or decision?)):ti,ab,kw | 747 |
| #46 | MeSH descriptor: [Data Mining] this term only | 20 |
| #47 | ((((data or text) NEAR/1 mining) or (data NEXT dashboard*)) NEAR/2 (introduc* or impact or effect? or evaluat* or implement* or decision?)):ti,ab,kw | 29 |
| #48 | MeSH descriptor: [Big Data] this term only | 5 |
| #49 | MeSH descriptor: [Algorithms] explode all trees | 4728 |
| #50 | ((algorithms or "artificial intelligence" or (machine* NEAR/1 (intelligen* or learn*)) or AI or "expert systems" or "fuzzy logic" or "deep learning" or robotics or "natural language processing") NEAR/2 (introduc* or impact or effect? or evaluat* or implement* or decision?)):ti,ab,kw | 506 |
| #51 | MeSH descriptor: [Electronic Data Processing] this term only | 92 |
| #52 | ((((electronic or automatic) NEAR/2 (data or information)) or (optical NEXT reader*) or (bar NEXT code*) or (qr NEXT code*)) NEAR/2 (introduc* or impact or effect? or evaluat* or implement* or decision?)):ti,ab,kw | 18 |
| #53 | (cloud NEAR/1 (comput* or process* or service* or stor*) NEAR/2 (introduc* or impact or effect? or evaluat* or implement* or decision?)):ti,ab,kw | 1 |
| #54 | #35 OR #36 OR #37 OR #38 OR #39 OR #40 OR #41 OR #42 OR #43 OR #44 OR #45 OR #46 OR #47 OR #48 OR #49 OR #50 OR #51 OR #52 OR #53 | 15116 |
| #55 | #54 NOT #34 | 9654 |

##

## Epistemonikos

| Database name | Epistemonikos |
| --- | --- |
| Database platform | Epistemonikos.org |
| Dates of database coverage | Complete database to search date |
| Date searched | 2022-06-20 |
| Searched by | JF |
| Number of results | Search 1: 5523 Search 2: 575 |
| EndNote import order | 5 |
| Number of results once duplicates removed | Search 1: 2729 Search 2: 305 |
| Search strategy notes | Searches filtered using:  Publication year 2009-2022 Publication type: systematic review |

Search 1

title:((education* AND (program* OR intervention* OR meeting* OR session* OR strateg* OR workshop* OR visit* OR method* OR material*)) OR (behavio* AND intervention*) OR (leaflet* OR booklet* OR poster OR posters) OR ((written OR printed OR oral OR campaign*) AND information) OR outreach OR ((opinion OR education* OR influential) AND leader*) OR facilitator* OR "academic detailing" OR "consensus conference*" OR "practice guideline*" OR (guideline* AND (introduc* OR issu* OR impact OR effect* OR disseminat* OR distribut*)) OR ((effect* OR impact OR evaluat* OR introduc* OR compar*) AND "training program*") OR reminder* OR (recall AND system*) OR prompter* OR prompting OR algorith* OR "chart review*" OR ((effect* OR impact OR records OR chart*) AND audit) OR marketing OR ((effect* OR impact OR evaluat* OR introduc* OR compara*) AND ("prevent* program*" OR "screening program*")) OR ((introduc* OR impact OR effect* OR implement* OR computer*) AND protocol*) OR (computer* AND (dosage OR dosing OR diagnosis OR therapy OR decision*)))

Search 2

(title:((digital* OR non-digital* OR cyber* OR computer* OR computing OR ((information OR communicat*) AND technolog*) or ICT OR (technolo* AND (advance* OR develop* OR new OR innovat* OR emerging OR transfer* OR licens*)) OR ((mobile OR cell OR cellular) AND (phone* OR telephone* OR handset* OR device*)) OR "text messag*" OR SMS OR texting OR smartphone OR "smart phone" OR ((handheld OR hand-held OR mobile) AND device*) OR "mobile handset*" OR software OR ((data OR text) AND mining) OR "data dashboard*" OR algorithms OR "artificial intelligence" OR (machine* AND (intelligen* OR learn*)) OR AI OR "expert systems" OR "fuzzy logic" OR "deep learning" OR robotics OR "natural language processing" OR ((electronic OR automatic) AND (data OR information)) OR "optical reader*" OR "bar code*" OR "qr code*") AND (introduc* or impact or effect* or evaluat* or implement* or decision*)) ) NOT (title:((education* AND (program* OR intervention* OR meeting* OR session* OR strateg* OR workshop* OR visit* OR method* OR material*)) OR (behavio* AND intervention*) OR (leaflet* OR booklet* OR poster OR posters) OR ((written OR printed OR oral OR campaign*) AND information) OR outreach OR ((opinion OR education* OR influential) AND leader*) OR facilitator* OR "academic detailing" OR "consensus conference*" OR "practice guideline*" OR (guideline* AND (introduc* OR issu* OR impact OR effect* OR disseminat* OR distribut*)) OR ((effect* OR impact OR evaluat* OR introduc* OR compar*) AND "training program*") OR reminder* OR (recall AND system*) OR prompter* OR prompting OR algorith* OR "chart review*" OR ((effect* OR impact OR records OR chart*) AND audit) OR marketing OR ((effect* OR impact OR evaluat* OR introduc* OR compara*) AND ("prevent* program*" OR "screening program*")) OR ((introduc* OR impact OR effect* OR implement* OR computer*) AND protocol*) OR (computer* AND (dosage OR dosing OR diagnosis OR therapy OR decision*))))
