# Supplementary material for: ‘It depends’: what 86 systematic reviews tell us about what strategies to use to support the use of research in clinical practice
Source: Implement Sci. 2024 Feb 19;19:15. doi: 10.1186/s13012-024-01337-z (PMC10875780; doi:10.1186/s13012-024-01337-z)
Supplement: Supplementary file 2 — Additional file 2: Appendix B. [file 13012_2024_1337_MOESM2_ESM.docx]

**Additional file 2**

**Appendix B:** Quality assessment checklist

REF ID: **Afari-Asiedu S. et al.** *Interventions to improve dispensing of antibiotics at the community level in low and middle income countries: a systematic review* (2022)

| **Questions** | | **NO**  Can´t tell or no info | **PARTIALLY**  Incomplete report | **YES** |
| --- | --- | --- | --- | --- |
| **1** | **Were the search methods used to find evidence (primary studies) on the primary question(s) stated?** |  | *Only the search terms are presented* | *Search terms are presented, and it is also explained how these search terms are combined (by “AND”, “OR”, etc.)* |
| **2** | **Was the search for evidence reasonably comprehensive?** |  | *Only Pubmed/Medline has been searched or*  *Search period is shorter than 10 years* |  |
| **3** | **Were the criteria used for deciding which studies to include in the review reported?** |  | *Only exclusion criteria are presented* | *Inclusion criteria are explicitly described* |
| **4** | **Was bias in the selection of articles avoided?** |  | *Only a part of the selection steps was completed by two reviewers independently* | *Both the initial selection (tiltle & abstract) and full-text reading were performed by two reviewers independently* |
| **5** | **Were the criteria used for assessing the validity of the studies that were reviewed reported?** |  |  | *The reviewer(s) used explicit assessment criteria or an existing assessment checklist* |
| **6** | **Was the validity of all of the studies referred to in the text assessed using appropriate criteria (either in selecting studies for inclusion or in analyzing the studies that are cited)?** |  | *Only the rigorousness of research designs is taken into account* | *Both in the selection phase and during analysis the methodological quality of the studies is taken into account using explicit criteria* |
| **7** | **Were the methods used to combine the findings of the relevant studies (to reach a conclusion) reported?** |  |  | *A “best evidence synthesis” is performed*  *Pooling of results has taken place*  *Results are presented on the basis of a an “evidence table”* |
| **8** | **Were the findings of the relevant studies combined appropriately relative to the primary question the review addresses?** | *No attempt made to combine findings*  *No statement regarding the inappropriateness of combining findings*  *A summary (general) estimate is given* *anywhere in the abstract, the discussion or the summary section of the paper but it is not reported how that estimate was derived* |  |  |
| **9** | **Were the conclusions made by the author(s) supported by the data and/ or analysis reported in the review?** |  |  | *Data (not just citations) should be reported that support the main conclusions* |
| **10** | **Overall, how would you rate the scientific quality of this review?**   \| Extensive flaws \|  \| Major flaws \|  \| Minor flaws \|  \| Minimal flaws \| \| --- \| --- \| --- \| --- \| --- \| --- \| --- \| \| 1 \| 2 \| 3 \| 4 \| 5 \| 6 \| 7 \|   *The score for question 10, the overall scientific quality, should be based on your answers to the first nine questions. The following guidelines can be used to assist with deriving a summary score: If the "can't tell" option is used one or more times on the preceding questions, a review is likely to have minor flaws at best and it is difficult to rule out major flaws (i.e. a score of 4 or lower) .If the "no” option is used on questions 2, 4, 6 or 8, the review is likely to have major flaws (i.e. a score of 3 or less, depending on the number and degree of the flaws).* | | | **6** |

REF ID: **Boonacker C.W.B. et al*.*** *Interventions in health care professionals to improve treatment in children with upper respiratory tract infections* (2010)

| **Questions** | | **NO**  Can´t tell or no info | **PARTIALLY**  Incomplete report | **YES** |
| --- | --- | --- | --- | --- |
| **1** | **Were the search methods used to find evidence (primary studies) on the primary question(s) stated?** |  | *Only the search terms are presented* | *Search terms are presented, and it is also explained how these search terms are combined (by “AND”, “OR”, etc.)* |
| **2** | **Was the search for evidence reasonably comprehensive?** |  | *Only Pubmed/Medline has been searched or*  *Search period is shorter than 10 years* |  |
| **3** | **Were the criteria used for deciding which studies to include in the review reported?** |  | *Only exclusion criteria are presented* | *Inclusion criteria are explicitly described* |
| **4** | **Was bias in the selection of articles avoided?** |  | *Only a part of the selection steps was completed by two reviewers independently* | *Both the initial selection (tiltle & abstract) and full-text reading were performed by two reviewers independently* |
| **5** | **Were the criteria used for assessing the validity of the studies that were reviewed reported?** |  |  | *The reviewer(s) used explicit assessment criteria or an existing assessment checklist* |
| **6** | **Was the validity of all of the studies referred to in the text assessed using appropriate criteria (either in selecting studies for inclusion or in analyzing the studies that are cited)?** |  | *Only the rigorousness of research designs is taken into account* | *Both in the selection phase and during analysis the methodological quality of the studies is taken into account using explicit criteria* |
| **7** | **Were the methods used to combine the findings of the relevant studies (to reach a conclusion) reported?** |  |  | *A “best evidence synthesis” is performed*  *Pooling of results has taken place*  *Results are presented on the basis of a an “evidence table”* |
| **8** | **Were the findings of the relevant studies combined appropriately relative to the primary question the review addresses?** | *No attempt made to combine findings*  *No statement regarding the inappropriateness of combining findings*  *A summary (general) estimate is given* *anywhere in the abstract, the discussion or the summary section of the paper but it is not reported how that estimate was derived* |  |  |
| **9** | **Were the conclusions made by the author(s) supported by the data and/ or analysis reported in the review?** |  |  | *Data (not just citations) should be reported that support the main conclusions* |
| **10** | **Overall, how would you rate the scientific quality of this review?**   \| Extensive flaws \|  \| Major flaws \|  \| Minor flaws \|  \| Minimal flaws \| \| --- \| --- \| --- \| --- \| --- \| --- \| --- \| \| 1 \| 2 \| 3 \| 4 \| 5 \| 6 \| 7 \|   *The score for question 10, the overall scientific quality, should be based on your answers to the first nine questions. The following guidelines can be used to assist with deriving a summary score: If the "can't tell" option is used one or more times on the preceding questions, a review is likely to have minor flaws at best and it is difficult to rule out major flaws (i.e. a score of 4 or lower) .If the "no” option is used on questions 2, 4, 6 or 8, the review is likely to have major flaws (i.e. a score of 3 or less, depending on the number and degree of the flaws).* | | | **6** |

REF ID: **De Angelis G. et al.** *Information and communication technologies for the dissemination of clinical practice guidelines to health professionals: a systematic review* (2016)

| **Questions** | | **NO**  Can´t tell or no info | **PARTIALLY**  Incomplete report | **YES** |
| --- | --- | --- | --- | --- |
| **1** | **Were the search methods used to find evidence (primary studies) on the primary question(s) stated?** |  | *Only the search terms are presented* | *Search terms are presented, and it is also explained how these search terms are combined (by “AND”, “OR”, etc.)* |
| **2** | **Was the search for evidence reasonably comprehensive?** |  | *Only Pubmed/Medline has been searched or*  *Search period is shorter than 10 years* |  |
| **3** | **Were the criteria used for deciding which studies to include in the review reported?** |  | *Only exclusion criteria are presented* | *Inclusion criteria are explicitly described* |
| **4** | **Was bias in the selection of articles avoided?** |  | *Only a part of the selection steps was completed by two reviewers independently* | *Both the initial selection (tiltle & abstract) and full-text reading were performed by two reviewers independently* |
| **5** | **Were the criteria used for assessing the validity of the studies that were reviewed reported?** |  |  | *The reviewer(s) used explicit assessment criteria or an existing assessment checklist* |
| **6** | **Was the validity of all of the studies referred to in the text assessed using appropriate criteria (either in selecting studies for inclusion or in analyzing the studies that are cited)?** |  | *Only the rigorousness of research designs is taken into account* | *Both in the selection phase and during analysis the methodological quality of the studies is taken into account using explicit criteria* |
| **7** | **Were the methods used to combine the findings of the relevant studies (to reach a conclusion) reported?** |  |  | *A “best evidence synthesis” is performed*  *Pooling of results has taken place*  *Results are presented on the basis of a an “evidence table”* |
| **8** | **Were the findings of the relevant studies combined appropriately relative to the primary question the review addresses?** | *No attempt made to combine findings*  *No statement regarding the inappropriateness of combining findings*  *A summary (general) estimate is given* *anywhere in the abstract, the discussion or the summary section of the paper but it is not reported how that estimate was derived* |  |  |
| **9** | **Were the conclusions made by the author(s) supported by the data and/ or analysis reported in the review?** |  |  | *Data (not just citations) should be reported that support the main conclusions* |
| **10** | **Overall, how would you rate the scientific quality of this review?**   \| Extensive flaws \|  \| Major flaws \|  \| Minor flaws \|  \| Minimal flaws \| \| --- \| --- \| --- \| --- \| --- \| --- \| --- \| \| 1 \| 2 \| 3 \| 4 \| 5 \| 6 \| 7 \|   *The score for question 10, the overall scientific quality, should be based on your answers to the first nine questions. The following guidelines can be used to assist with deriving a summary score: If the "can't tell" option is used one or more times on the preceding questions, a review is likely to have minor flaws at best and it is difficult to rule out major flaws (i.e. a score of 4 or lower) .If the "no” option is used on questions 2, 4, 6 or 8, the review is likely to have major flaws (i.e. a score of 3 or less, depending on the number and degree of the flaws).* | | | **6** |

REF ID: **Al Zoubi F.M. et al*.*** *The effectiveness of interventions designed to increase the uptake of clinical practice guidelines and best practices among musculoskeletal professionals: a systematic review* (2018)

| **Questions** | | **NO**  Can´t tell or no info | **PARTIALLY**  Incomplete report | **YES** |
| --- | --- | --- | --- | --- |
| **1** | **Were the search methods used to find evidence (primary studies) on the primary question(s) stated?** |  | *Only the search terms are presented* | *Search terms are presented, and it is also explained how these search terms are combined (by “AND”, “OR”, etc.)* |
| **2** | **Was the search for evidence reasonably comprehensive?** |  | *Only Pubmed/Medline has been searched or*  *Search period is shorter than 10 years* |  |
| **3** | **Were the criteria used for deciding which studies to include in the review reported?** |  | *Only exclusion criteria are presented* | *Inclusion criteria are explicitly described* |
| **4** | **Was bias in the selection of articles avoided?** |  | *Only a part of the selection steps was completed by two reviewers independently* | *Both the initial selection (tiltle & abstract) and full-text reading were performed by two reviewers independently* |
| **5** | **Were the criteria used for assessing the validity of the studies that were reviewed reported?** |  |  | *The reviewer(s) used explicit assessment criteria or an existing assessment checklist* |
| **6** | **Was the validity of all of the studies referred to in the text assessed using appropriate criteria (either in selecting studies for inclusion or in analyzing the studies that are cited)?** |  | *Only the rigorousness of research designs is taken into account* | *Both in the selection phase and during analysis the methodological quality of the studies is taken into account using explicit criteria* |
| **7** | **Were the methods used to combine the findings of the relevant studies (to reach a conclusion) reported?** |  |  | *A “best evidence synthesis” is performed*  *Pooling of results has taken place*  *Results are presented on the basis of a an “evidence table”* |
| **8** | **Were the findings of the relevant studies combined appropriately relative to the primary question the review addresses?** | *No attempt made to combine findings*  *No statement regarding the inappropriateness of combining findings*  *A summary (general) estimate is given* *anywhere in the abstract, the discussion or the summary section of the paper but it is not reported how that estimate was derived* |  |  |
| **9** | **Were the conclusions made by the author(s) supported by the data and/ or analysis reported in the review?** |  |  | *Data (not just citations) should be reported that support the main conclusions* |
| **10** | **Overall, how would you rate the scientific quality of this review?**   \| Extensive flaws \|  \| Major flaws \|  \| Minor flaws \|  \| Minimal flaws \| \| --- \| --- \| --- \| --- \| --- \| --- \| --- \| \| 1 \| 2 \| 3 \| 4 \| 5 \| 6 \| 7 \|   *The score for question 10, the overall scientific quality, should be based on your answers to the first nine questions. The following guidelines can be used to assist with deriving a summary score: If the "can't tell" option is used one or more times on the preceding questions, a review is likely to have minor flaws at best and it is difficult to rule out major flaws (i.e. a score of 4 or lower) .If the "no” option is used on questions 2, 4, 6 or 8, the review is likely to have major flaws (i.e. a score of 3 or less, depending on the number and degree of the flaws).* | | | **7** |

REF ID: **Ariyo P. et al.** *Implementation strategies to reduce surgical site infections: a systematic review* (2019)

| **Questions** | | **NO**  Can´t tell or no info | **PARTIALLY**  Incomplete report | **YES** |
| --- | --- | --- | --- | --- |
| **1** | **Were the search methods used to find evidence (primary studies) on the primary question(s) stated?** |  | *Only the search terms are presented* | *Search terms are presented, and it is also explained how these search terms are combined (by “AND”, “OR”, etc.)* |
| **2** | **Was the search for evidence reasonably comprehensive?** |  | *Only Pubmed/Medline has been searched or*  *Search period is shorter than 10 years* |  |
| **3** | **Were the criteria used for deciding which studies to include in the review reported?** |  | *Only exclusion criteria are presented* | *Inclusion criteria are explicitly described* |
| **4** | **Was bias in the selection of articles avoided?** |  | *Only a part of the selection steps was completed by two reviewers independently* | *Both the initial selection (tiltle & abstract) and full-text reading were performed by two reviewers independently* |
| **5** | **Were the criteria used for assessing the validity of the studies that were reviewed reported?** |  |  | *The reviewer(s) used explicit assessment criteria or an existing assessment checklist* |
| **6** | **Was the validity of all of the studies referred to in the text assessed using appropriate criteria (either in selecting studies for inclusion or in analyzing the studies that are cited)?** |  | *Only the rigorousness of research designs is taken into account* | *Both in the selection phase and during analysis the methodological quality of the studies is taken into account using explicit criteria* |
| **7** | **Were the methods used to combine the findings of the relevant studies (to reach a conclusion) reported?** |  |  | *A “best evidence synthesis” is performed*  *Pooling of results has taken place*  *Results are presented on the basis of a an “evidence table”* |
| **8** | **Were the findings of the relevant studies combined appropriately relative to the primary question the review addresses?** | *No attempt made to combine findings*  *No statement regarding the inappropriateness of combining findings*  *A summary (general) estimate is given* *anywhere in the abstract, the discussion or the summary section of the paper but it is not reported how that estimate was derived* |  |  |
| **9** | **Were the conclusions made by the author(s) supported by the data and/ or analysis reported in the review?** |  |  | *Data (not just citations) should be reported that support the main conclusions* |
| **10** | **Overall, how would you rate the scientific quality of this review?**   \| Extensive flaws \|  \| Major flaws \|  \| Minor flaws \|  \| Minimal flaws \| \| --- \| --- \| --- \| --- \| --- \| --- \| --- \| \| 1 \| 2 \| 3 \| 4 \| 5 \| 6 \| 7 \|   *The score for question 10, the overall scientific quality, should be based on your answers to the first nine questions. The following guidelines can be used to assist with deriving a summary score: If the "can't tell" option is used one or more times on the preceding questions, a review is likely to have minor flaws at best and it is difficult to rule out major flaws (i.e. a score of 4 or lower) .If the "no” option is used on questions 2, 4, 6 or 8, the review is likely to have major flaws (i.e. a score of 3 or less, depending on the number and degree of the flaws).* | | | **5** |

REF ID: **Borgert M.J., Goossens A. & Dongelmans D.A.** *What are effective strategies for the implementation of care bundles on ICUs: a systematic review* (2015)

| **Questions** | | **NO**  Can´t tell or no info | **PARTIALLY**  Incomplete report | **YES** |
| --- | --- | --- | --- | --- |
| **1** | **Were the search methods used to find evidence (primary studies) on the primary question(s) stated?** |  | *Only the search terms are presented* | *Search terms are presented, and it is also explained how these search terms are combined (by “AND”, “OR”, etc.)* |
| **2** | **Was the search for evidence reasonably comprehensive?** |  | *Only Pubmed/Medline has been searched or*  *Search period is shorter than 10 years* |  |
| **3** | **Were the criteria used for deciding which studies to include in the review reported?** |  | *Only exclusion criteria are presented* | *Inclusion criteria are explicitly described* |
| **4** | **Was bias in the selection of articles avoided?** |  | *Only a part of the selection steps was completed by two reviewers independently* | *Both the initial selection (tiltle & abstract) and full-text reading were performed by two reviewers independently* |
| **5** | **Were the criteria used for assessing the validity of the studies that were reviewed reported?** |  |  | *The reviewer(s) used explicit assessment criteria or an existing assessment checklist* |
| **6** | **Was the validity of all of the studies referred to in the text assessed using appropriate criteria (either in selecting studies for inclusion or in analyzing the studies that are cited)?** |  | *Only the rigorousness of research designs is taken into account* | *Both in the selection phase and during analysis the methodological quality of the studies is taken into account using explicit criteria* |
| **7** | **Were the methods used to combine the findings of the relevant studies (to reach a conclusion) reported?** |  |  | *A “best evidence synthesis” is performed*  *Pooling of results has taken place*  *Results are presented on the basis of a an “evidence table”* |
| **8** | **Were the findings of the relevant studies combined appropriately relative to the primary question the review addresses?** | *No attempt made to combine findings*  *No statement regarding the inappropriateness of combining findings*  *A summary (general) estimate is given* *anywhere in the abstract, the discussion or the summary section of the paper but it is not reported how that estimate was derived* |  |  |
| **9** | **Were the conclusions made by the author(s) supported by the data and/ or analysis reported in the review?** |  |  | *Data (not just citations) should be reported that support the main conclusions* |
| **10** | **Overall, how would you rate the scientific quality of this review?**   \| Extensive flaws \|  \| Major flaws \|  \| Minor flaws \|  \| Minimal flaws \| \| --- \| --- \| --- \| --- \| --- \| --- \| --- \| \| 1 \| 2 \| 3 \| 4 \| 5 \| 6 \| 7 \|   *The score for question 10, the overall scientific quality, should be based on your answers to the first nine questions. The following guidelines can be used to assist with deriving a summary score: If the "can't tell" option is used one or more times on the preceding questions, a review is likely to have minor flaws at best and it is difficult to rule out major flaws (i.e. a score of 4 or lower) .If the "no” option is used on questions 2, 4, 6 or 8, the review is likely to have major flaws (i.e. a score of 3 or less, depending on the number and degree of the flaws).* | | | **7** |

REF ID: **Cahill L. et al.** *Implementation interventions to promote the uptake of evidence- based practices in stroke rehabilitation (Review)* (2020)

| **Questions** | | **NO**  Can´t tell or no info | **PARTIALLY**  Incomplete report | **YES** |
| --- | --- | --- | --- | --- |
| **1** | **Were the search methods used to find evidence (primary studies) on the primary question(s) stated?** |  | *Only the search terms are presented* | *Search terms are presented, and it is also explained how these search terms are combined (by “AND”, “OR”, etc.)* |
| **2** | **Was the search for evidence reasonably comprehensive?** |  | *Only Pubmed/Medline has been searched or*  *Search period is shorter than 10 years* |  |
| **3** | **Were the criteria used for deciding which studies to include in the review reported?** |  | *Only exclusion criteria are presented* | *Inclusion criteria are explicitly described* |
| **4** | **Was bias in the selection of articles avoided?** |  | *Only a part of the selection steps was completed by two reviewers independently* | *Both the initial selection (tiltle & abstract) and full-text reading were performed by two reviewers independently* |
| **5** | **Were the criteria used for assessing the validity of the studies that were reviewed reported?** |  |  | *The reviewer(s) used explicit assessment criteria or an existing assessment checklist* |
| **6** | **Was the validity of all of the studies referred to in the text assessed using appropriate criteria (either in selecting studies for inclusion or in analyzing the studies that are cited)?** |  | *Only the rigorousness of research designs is taken into account* | *Both in the selection phase and during analysis the methodological quality of the studies is taken into account using explicit criteria* |
| **7** | **Were the methods used to combine the findings of the relevant studies (to reach a conclusion) reported?** |  |  | *A “best evidence synthesis” is performed*  *Pooling of results has taken place*  *Results are presented on the basis of a an “evidence table”* |
| **8** | **Were the findings of the relevant studies combined appropriately relative to the primary question the review addresses?** | *No attempt made to combine findings*  *No statement regarding the inappropriateness of combining findings*  *A summary (general) estimate is given* *anywhere in the abstract, the discussion or the summary section of the paper but it is not reported how that estimate was derived* |  |  |
| **9** | **Were the conclusions made by the author(s) supported by the data and/ or analysis reported in the review?** |  |  | *Data (not just citations) should be reported that support the main conclusions* |
| **10** | **Overall, how would you rate the scientific quality of this review?**   \| Extensive flaws \|  \| Major flaws \|  \| Minor flaws \|  \| Minimal flaws \| \| --- \| --- \| --- \| --- \| --- \| --- \| --- \| \| 1 \| 2 \| 3 \| 4 \| 5 \| 6 \| 7 \|   *The score for question 10, the overall scientific quality, should be based on your answers to the first nine questions. The following guidelines can be used to assist with deriving a summary score: If the "can't tell" option is used one or more times on the preceding questions, a review is likely to have minor flaws at best and it is difficult to rule out major flaws (i.e. a score of 4 or lower) .If the "no” option is used on questions 2, 4, 6 or 8, the review is likely to have major flaws (i.e. a score of 3 or less, depending on the number and degree of the flaws).* | | | **7** |

REF ID: **Pedersen E.R. et al.** *Elusive search for effective provider interventions: a systematic review of provider interventions to increase adherence to evidence-based treatment for depression* (2018)

| **Questions** | | **NO**  Can´t tell or no info | **PARTIALLY**  Incomplete report | **YES** |
| --- | --- | --- | --- | --- |
| **1** | **Were the search methods used to find evidence (primary studies) on the primary question(s) stated?** |  | *Only the search terms are presented* | *Search terms are presented, and it is also explained how these search terms are combined (by “AND”, “OR”, etc.)* |
| **2** | **Was the search for evidence reasonably comprehensive?** |  | *Only Pubmed/Medline has been searched or*  *Search period is shorter than 10 years* |  |
| **3** | **Were the criteria used for deciding which studies to include in the review reported?** |  | *Only exclusion criteria are presented* | *Inclusion criteria are explicitly described* |
| **4** | **Was bias in the selection of articles avoided?** |  | *Only a part of the selection steps was completed by two reviewers independently* | *Both the initial selection (tiltle & abstract) and full-text reading were performed by two reviewers independently* |
| **5** | **Were the criteria used for assessing the validity of the studies that were reviewed reported?** |  |  | *The reviewer(s) used explicit assessment criteria or an existing assessment checklist* |
| **6** | **Was the validity of all of the studies referred to in the text assessed using appropriate criteria (either in selecting studies for inclusion or in analyzing the studies that are cited)?** |  | *Only the rigorousness of research designs is taken into account* | *Both in the selection phase and during analysis the methodological quality of the studies is taken into account using explicit criteria* |
| **7** | **Were the methods used to combine the findings of the relevant studies (to reach a conclusion) reported?** |  |  | *A “best evidence synthesis” is performed*  *Pooling of results has taken place*  *Results are presented on the basis of a an “evidence table”* |
| **8** | **Were the findings of the relevant studies combined appropriately relative to the primary question the review addresses?** | *No attempt made to combine findings*  *No statement regarding the inappropriateness of combining findings*  *A summary (general) estimate is given* *anywhere in the abstract, the discussion or the summary section of the paper but it is not reported how that estimate was derived* |  |  |
| **9** | **Were the conclusions made by the author(s) supported by the data and/ or analysis reported in the review?** |  |  | *Data (not just citations) should be reported that support the main conclusions* |
| **10** | **Overall, how would you rate the scientific quality of this review?**   \| Extensive flaws \|  \| Major flaws \|  \| Minor flaws \|  \| Minimal flaws \| \| --- \| --- \| --- \| --- \| --- \| --- \| --- \| \| 1 \| 2 \| 3 \| 4 \| 5 \| 6 \| 7 \|   *The score for question 10, the overall scientific quality, should be based on your answers to the first nine questions. The following guidelines can be used to assist with deriving a summary score: If the "can't tell" option is used one or more times on the preceding questions, a review is likely to have minor flaws at best and it is difficult to rule out major flaws (i.e. a score of 4 or lower) .If the "no” option is used on questions 2, 4, 6 or 8, the review is likely to have major flaws (i.e. a score of 3 or less, depending on the number and degree of the flaws).* | | | **7** |

REF ID: **Yamada J. et al.** *The effectiveness of toolkits as knowledge translation strategies for integrating evidence into clinical care: a systematic review* (2015)

| **Questions** | | **NO**  Can´t tell or no info | **PARTIALLY**  Incomplete report | **YES** |
| --- | --- | --- | --- | --- |
| **1** | **Were the search methods used to find evidence (primary studies) on the primary question(s) stated?** |  | *Only the search terms are presented* | *Search terms are presented, and it is also explained how these search terms are combined (by “AND”, “OR”, etc.)* |
| **2** | **Was the search for evidence reasonably comprehensive?** |  | *Only Pubmed/Medline has been searched or*  *Search period is shorter than 10 years* |  |
| **3** | **Were the criteria used for deciding which studies to include in the review reported?** |  | *Only exclusion criteria are presented* | *Inclusion criteria are explicitly described* |
| **4** | **Was bias in the selection of articles avoided?** |  | *Only a part of the selection steps was completed by two reviewers independently* | *Both the initial selection (tiltle & abstract) and full-text reading were performed by two reviewers independently* |
| **5** | **Were the criteria used for assessing the validity of the studies that were reviewed reported?** |  |  | *The reviewer(s) used explicit assessment criteria or an existing assessment checklist* |
| **6** | **Was the validity of all of the studies referred to in the text assessed using appropriate criteria (either in selecting studies for inclusion or in analyzing the studies that are cited)?** |  | *Only the rigorousness of research designs is taken into account* | *Both in the selection phase and during analysis the methodological quality of the studies is taken into account using explicit criteria* |
| **7** | **Were the methods used to combine the findings of the relevant studies (to reach a conclusion) reported?** |  |  | *A “best evidence synthesis” is performed*  *Pooling of results has taken place*  *Results are presented on the basis of a an “evidence table”* |
| **8** | **Were the findings of the relevant studies combined appropriately relative to the primary question the review addresses?** | *No attempt made to combine findings*  *No statement regarding the inappropriateness of combining findings*  *A summary (general) estimate is given* *anywhere in the abstract, the discussion or the summary section of the paper but it is not reported how that estimate was derived* |  |  |
| **9** | **Were the conclusions made by the author(s) supported by the data and/ or analysis reported in the review?** |  |  | *Data (not just citations) should be reported that support the main conclusions* |
| **10** | **Overall, how would you rate the scientific quality of this review?**   \| Extensive flaws \|  \| Major flaws \|  \| Minor flaws \|  \| Minimal flaws \| \| --- \| --- \| --- \| --- \| --- \| --- \| --- \| \| 1 \| 2 \| 3 \| 4 \| 5 \| 6 \| 7 \|   *The score for question 10, the overall scientific quality, should be based on your answers to the first nine questions. The following guidelines can be used to assist with deriving a summary score: If the "can't tell" option is used one or more times on the preceding questions, a review is likely to have minor flaws at best and it is difficult to rule out major flaws (i.e. a score of 4 or lower) .If the "no” option is used on questions 2, 4, 6 or 8, the review is likely to have major flaws (i.e. a score of 3 or less, depending on the number and degree of the flaws).* | | | **7** |

REF ID: **Brown A. et al.** *Effectiveness of technology-enabled knowledge translation strategies in improving the use of research in public health: systematic review* (2020)

| **Questions** | | **NO**  Can´t tell or no info | **PARTIALLY**  Incomplete report | **YES** |
| --- | --- | --- | --- | --- |
| **1** | **Were the search methods used to find evidence (primary studies) on the primary question(s) stated?** |  | *Only the search terms are presented* | *Search terms are presented, and it is also explained how these search terms are combined (by “AND”, “OR”, etc.)* |
| **2** | **Was the search for evidence reasonably comprehensive?** |  | *Only Pubmed/Medline has been searched or*  *Search period is shorter than 10 years* |  |
| **3** | **Were the criteria used for deciding which studies to include in the review reported?** |  | *Only exclusion criteria are presented* | *Inclusion criteria are explicitly described* |
| **4** | **Was bias in the selection of articles avoided?** |  | *Only a part of the selection steps was completed by two reviewers independently* | *Both the initial selection (tiltle & abstract) and full-text reading were performed by two reviewers independently* |
| **5** | **Were the criteria used for assessing the validity of the studies that were reviewed reported?** |  |  | *The reviewer(s) used explicit assessment criteria or an existing assessment checklist* |
| **6** | **Was the validity of all of the studies referred to in the text assessed using appropriate criteria (either in selecting studies for inclusion or in analyzing the studies that are cited)?** |  | *Only the rigorousness of research designs is taken into account* | *Both in the selection phase and during analysis the methodological quality of the studies is taken into account using explicit criteria* |
| **7** | **Were the methods used to combine the findings of the relevant studies (to reach a conclusion) reported?** |  |  | *A “best evidence synthesis” is performed*  *Pooling of results has taken place*  *Results are presented on the basis of a an “evidence table”* |
| **8** | **Were the findings of the relevant studies combined appropriately relative to the primary question the review addresses?** | *No attempt made to combine findings*  *No statement regarding the inappropriateness of combining findings*  *A summary (general) estimate is given* *anywhere in the abstract, the discussion or the summary section of the paper but it is not reported how that estimate was derived* |  |  |
| **9** | **Were the conclusions made by the author(s) supported by the data and/ or analysis reported in the review?** |  |  | *Data (not just citations) should be reported that support the main conclusions* |
| **10** | **Overall, how would you rate the scientific quality of this review?**   \| Extensive flaws \|  \| Major flaws \|  \| Minor flaws \|  \| Minimal flaws \| \| --- \| --- \| --- \| --- \| --- \| --- \| --- \| \| 1 \| 2 \| 3 \| 4 \| 5 \| 6 \| 7 \|   *The score for question 10, the overall scientific quality, should be based on your answers to the first nine questions. The following guidelines can be used to assist with deriving a summary score: If the "can't tell" option is used one or more times on the preceding questions, a review is likely to have minor flaws at best and it is difficult to rule out major flaws (i.e. a score of 4 or lower) .If the "no” option is used on questions 2, 4, 6 or 8, the review is likely to have major flaws (i.e. a score of 3 or less, depending on the number and degree of the flaws).* | | | **7** |

REF ID: **Grudniewicz A. et al.** *What is the effectiveness of printed educational materials on primary care physician knowledge, behaviour, and patient outcomes: a systematic review and meta-analyses* (2015)

| **Questions** | | **NO**  Can´t tell or no info | **PARTIALLY**  Incomplete report | **YES** |
| --- | --- | --- | --- | --- |
| **1** | **Were the search methods used to find evidence (primary studies) on the primary question(s) stated?** |  | *Only the search terms are presented* | *Search terms are presented, and it is also explained how these search terms are combined (by “AND”, “OR”, etc.)* |
| **2** | **Was the search for evidence reasonably comprehensive?** |  | *Only Pubmed/Medline has been searched or*  *Search period is shorter than 10 years* |  |
| **3** | **Were the criteria used for deciding which studies to include in the review reported?** |  | *Only exclusion criteria are presented* | *Inclusion criteria are explicitly described* |
| **4** | **Was bias in the selection of articles avoided?** |  | *Only a part of the selection steps was completed by two reviewers independently* | *Both the initial selection (tiltle & abstract) and full-text reading were performed by two reviewers independently* |
| **5** | **Were the criteria used for assessing the validity of the studies that were reviewed reported?** |  |  | *The reviewer(s) used explicit assessment criteria or an existing assessment checklist* |
| **6** | **Was the validity of all of the studies referred to in the text assessed using appropriate criteria (either in selecting studies for inclusion or in analyzing the studies that are cited)?** |  | *Only the rigorousness of research designs is taken into account* | *Both in the selection phase and during analysis the methodological quality of the studies is taken into account using explicit criteria* |
| **7** | **Were the methods used to combine the findings of the relevant studies (to reach a conclusion) reported?** |  |  | *A “best evidence synthesis” is performed*  *Pooling of results has taken place*  *Results are presented on the basis of a an “evidence table”* |
| **8** | **Were the findings of the relevant studies combined appropriately relative to the primary question the review addresses?** | *No attempt made to combine findings*  *No statement regarding the inappropriateness of combining findings*  *A summary (general) estimate is given* *anywhere in the abstract, the discussion or the summary section of the paper but it is not reported how that estimate was derived* |  |  |
| **9** | **Were the conclusions made by the author(s) supported by the data and/ or analysis reported in the review?** |  |  | *Data (not just citations) should be reported that support the main conclusions* |
| **10** | **Overall, how would you rate the scientific quality of this review?**   \| Extensive flaws \|  \| Major flaws \|  \| Minor flaws \|  \| Minimal flaws \| \| --- \| --- \| --- \| --- \| --- \| --- \| --- \| \| 1 \| 2 \| 3 \| 4 \| 5 \| 6 \| 7 \|   *The score for question 10, the overall scientific quality, should be based on your answers to the first nine questions. The following guidelines can be used to assist with deriving a summary score: If the "can't tell" option is used one or more times on the preceding questions, a review is likely to have minor flaws at best and it is difficult to rule out major flaws (i.e. a score of 4 or lower) .If the "no” option is used on questions 2, 4, 6 or 8, the review is likely to have major flaws (i.e. a score of 3 or less, depending on the number and degree of the flaws).* | | | **7** |

REF ID: **Jenkins H.J. et al.** *Effectiveness of interventions designed to reduce the use of imaging for low-back pain: a systematic review* (2015)

| **Questions** | | **NO**  Can´t tell or no info | **PARTIALLY**  Incomplete report | **YES** |
| --- | --- | --- | --- | --- |
| **1** | **Were the search methods used to find evidence (primary studies) on the primary question(s) stated?** |  | *Only the search terms are presented* | *Search terms are presented, and it is also explained how these search terms are combined (by “AND”, “OR”, etc.)* |
| **2** | **Was the search for evidence reasonably comprehensive?** |  | *Only Pubmed/Medline has been searched or*  *Search period is shorter than 10 years* |  |
| **3** | **Were the criteria used for deciding which studies to include in the review reported?** |  | *Only exclusion criteria are presented* | *Inclusion criteria are explicitly described* |
| **4** | **Was bias in the selection of articles avoided?** |  | *Only a part of the selection steps was completed by two reviewers independently* | *Both the initial selection (tiltle & abstract) and full-text reading were performed by two reviewers independently* |
| **5** | **Were the criteria used for assessing the validity of the studies that were reviewed reported?** |  |  | *The reviewer(s) used explicit assessment criteria or an existing assessment checklist* |
| **6** | **Was the validity of all of the studies referred to in the text assessed using appropriate criteria (either in selecting studies for inclusion or in analyzing the studies that are cited)?** |  | *Only the rigorousness of research designs is taken into account* | *Both in the selection phase and during analysis the methodological quality of the studies is taken into account using explicit criteria* |
| **7** | **Were the methods used to combine the findings of the relevant studies (to reach a conclusion) reported?** |  |  | *A “best evidence synthesis” is performed*  *Pooling of results has taken place*  *Results are presented on the basis of a an “evidence table”* |
| **8** | **Were the findings of the relevant studies combined appropriately relative to the primary question the review addresses?** | *No attempt made to combine findings*  *No statement regarding the inappropriateness of combining findings*  *A summary (general) estimate is given* *anywhere in the abstract, the discussion or the summary section of the paper but it is not reported how that estimate was derived* |  |  |
| **9** | **Were the conclusions made by the author(s) supported by the data and/ or analysis reported in the review?** |  |  | *Data (not just citations) should be reported that support the main conclusions* |
| **10** | **Overall, how would you rate the scientific quality of this review?**   \| Extensive flaws \|  \| Major flaws \|  \| Minor flaws \|  \| Minimal flaws \| \| --- \| --- \| --- \| --- \| --- \| --- \| --- \| \| 1 \| 2 \| 3 \| 4 \| 5 \| 6 \| 7 \|   *The score for question 10, the overall scientific quality, should be based on your answers to the first nine questions. The following guidelines can be used to assist with deriving a summary score: If the "can't tell" option is used one or more times on the preceding questions, a review is likely to have minor flaws at best and it is difficult to rule out major flaws (i.e. a score of 4 or lower) .If the "no” option is used on questions 2, 4, 6 or 8, the review is likely to have major flaws (i.e. a score of 3 or less, depending on the number and degree of the flaws).* | | | **6** |

REF ID: **Koota E., Kääriäinen M. & Melender H-L.** *Educational interventions promoting evidence-based practice among emergency nurses: A systematic review* (2018)

| **Questions** | | **NO**  Can´t tell or no info | **PARTIALLY**  Incomplete report | **YES** |
| --- | --- | --- | --- | --- |
| **1** | **Were the search methods used to find evidence (primary studies) on the primary question(s) stated?** |  | *Only the search terms are presented* | *Search terms are presented, and it is also explained how these search terms are combined (by “AND”, “OR”, etc.)* |
| **2** | **Was the search for evidence reasonably comprehensive?** |  | *Only Pubmed/Medline has been searched or*  *Search period is shorter than 10 years* |  |
| **3** | **Were the criteria used for deciding which studies to include in the review reported?** |  | *Only exclusion criteria are presented* | *Inclusion criteria are explicitly described* |
| **4** | **Was bias in the selection of articles avoided?** |  | *Only a part of the selection steps was completed by two reviewers independently* | *Both the initial selection (tiltle & abstract) and full-text reading were performed by two reviewers independently* |
| **5** | **Were the criteria used for assessing the validity of the studies that were reviewed reported?** |  |  | *The reviewer(s) used explicit assessment criteria or an existing assessment checklist* |
| **6** | **Was the validity of all of the studies referred to in the text assessed using appropriate criteria (either in selecting studies for inclusion or in analyzing the studies that are cited)?** |  | *Only the rigorousness of research designs is taken into account* | *Both in the selection phase and during analysis the methodological quality of the studies is taken into account using explicit criteria* |
| **7** | **Were the methods used to combine the findings of the relevant studies (to reach a conclusion) reported?** |  |  | *A “best evidence synthesis” is performed*  *Pooling of results has taken place*  *Results are presented on the basis of a an “evidence table”* |
| **8** | **Were the findings of the relevant studies combined appropriately relative to the primary question the review addresses?** | *No attempt made to combine findings*  *No statement regarding the inappropriateness of combining findings*  *A summary (general) estimate is given* *anywhere in the abstract, the discussion or the summary section of the paper but it is not reported how that estimate was derived* |  |  |
| **9** | **Were the conclusions made by the author(s) supported by the data and/ or analysis reported in the review?** |  |  | *Data (not just citations) should be reported that support the main conclusions* |
| **10** | **Overall, how would you rate the scientific quality of this review?**   \| Extensive flaws \|  \| Major flaws \|  \| Minor flaws \|  \| Minimal flaws \| \| --- \| --- \| --- \| --- \| --- \| --- \| --- \| \| 1 \| 2 \| 3 \| 4 \| 5 \| 6 \| 7 \|   *The score for question 10, the overall scientific quality, should be based on your answers to the first nine questions. The following guidelines can be used to assist with deriving a summary score: If the "can't tell" option is used one or more times on the preceding questions, a review is likely to have minor flaws at best and it is difficult to rule out major flaws (i.e. a score of 4 or lower) .If the "no” option is used on questions 2, 4, 6 or 8, the review is likely to have major flaws (i.e. a score of 3 or less, depending on the number and degree of the flaws).* | | | **7** |

REF ID: **Wu Y. et al.** *Do educational interventions aimed at nurses to support the implementation of evidence-based practice improve patient outcomes? A systematic review* (2018)

| **Questions** | | **NO**  Can´t tell or no info | **PARTIALLY**  Incomplete report | **YES** |
| --- | --- | --- | --- | --- |
| **1** | **Were the search methods used to find evidence (primary studies) on the primary question(s) stated?** |  | *Only the search terms are presented* | *Search terms are presented, and it is also explained how these search terms are combined (by “AND”, “OR”, etc.)* |
| **2** | **Was the search for evidence reasonably comprehensive?** |  | *Only Pubmed/Medline has been searched or*  *Search period is shorter than 10 years* |  |
| **3** | **Were the criteria used for deciding which studies to include in the review reported?** |  | *Only exclusion criteria are presented* | *Inclusion criteria are explicitly described* |
| **4** | **Was bias in the selection of articles avoided?** |  | *Only a part of the selection steps was completed by two reviewers independently* | *Both the initial selection (tiltle & abstract) and full-text reading were performed by two reviewers independently* |
| **5** | **Were the criteria used for assessing the validity of the studies that were reviewed reported?** |  |  | *The reviewer(s) used explicit assessment criteria or an existing assessment checklist* |
| **6** | **Was the validity of all of the studies referred to in the text assessed using appropriate criteria (either in selecting studies for inclusion or in analyzing the studies that are cited)?** |  | *Only the rigorousness of research designs is taken into account* | *Both in the selection phase and during analysis the methodological quality of the studies is taken into account using explicit criteria* |
| **7** | **Were the methods used to combine the findings of the relevant studies (to reach a conclusion) reported?** |  |  | *A “best evidence synthesis” is performed*  *Pooling of results has taken place*  *Results are presented on the basis of a an “evidence table”* |
| **8** | **Were the findings of the relevant studies combined appropriately relative to the primary question the review addresses?** | *No attempt made to combine findings*  *No statement regarding the inappropriateness of combining findings*  *A summary (general) estimate is given* *anywhere in the abstract, the discussion or the summary section of the paper but it is not reported how that estimate was derived* |  |  |
| **9** | **Were the conclusions made by the author(s) supported by the data and/ or analysis reported in the review?** |  |  | *Data (not just citations) should be reported that support the main conclusions* |
| **10** | **Overall, how would you rate the scientific quality of this review?**   \| Extensive flaws \|  \| Major flaws \|  \| Minor flaws \|  \| Minimal flaws \| \| --- \| --- \| --- \| --- \| --- \| --- \| --- \| \| 1 \| 2 \| 3 \| 4 \| 5 \| 6 \| 7 \|   *The score for question 10, the overall scientific quality, should be based on your answers to the first nine questions. The following guidelines can be used to assist with deriving a summary score: If the "can't tell" option is used one or more times on the preceding questions, a review is likely to have minor flaws at best and it is difficult to rule out major flaws (i.e. a score of 4 or lower) .If the "no” option is used on questions 2, 4, 6 or 8, the review is likely to have major flaws (i.e. a score of 3 or less, depending on the number and degree of the flaws).* | | | **7** |

REF ID: **Arditi C. et al.** *Computer-generated reminders delivered on paper to healthcare professionals: effects on professional practice and healthcare outcomes* (2017)

| **Questions** | | **NO**  Can´t tell or no info | **PARTIALLY**  Incomplete report | **YES** |
| --- | --- | --- | --- | --- |
| **1** | **Were the search methods used to find evidence (primary studies) on the primary question(s) stated?** |  | *Only the search terms are presented* | *Search terms are presented, and it is also explained how these search terms are combined (by “AND”, “OR”, etc.)* |
| **2** | **Was the search for evidence reasonably comprehensive?** |  | *Only Pubmed/Medline has been searched or*  *Search period is shorter than 10 years* |  |
| **3** | **Were the criteria used for deciding which studies to include in the review reported?** |  | *Only exclusion criteria are presented* | *Inclusion criteria are explicitly described* |
| **4** | **Was bias in the selection of articles avoided?** |  | *Only a part of the selection steps was completed by two reviewers independently* | *Both the initial selection (tiltle & abstract) and full-text reading were performed by two reviewers independently* |
| **5** | **Were the criteria used for assessing the validity of the studies that were reviewed reported?** |  |  | *The reviewer(s) used explicit assessment criteria or an existing assessment checklist* |
| **6** | **Was the validity of all of the studies referred to in the text assessed using appropriate criteria (either in selecting studies for inclusion or in analyzing the studies that are cited)?** |  | *Only the rigorousness of research designs is taken into account* | *Both in the selection phase and during analysis the methodological quality of the studies is taken into account using explicit criteria* |
| **7** | **Were the methods used to combine the findings of the relevant studies (to reach a conclusion) reported?** |  |  | *A “best evidence synthesis” is performed*  *Pooling of results has taken place*  *Results are presented on the basis of a an “evidence table”* |
| **8** | **Were the findings of the relevant studies combined appropriately relative to the primary question the review addresses?** | *No attempt made to combine findings*  *No statement regarding the inappropriateness of combining findings*  *A summary (general) estimate is given* *anywhere in the abstract, the discussion or the summary section of the paper but it is not reported how that estimate was derived* |  |  |
| **9** | **Were the conclusions made by the author(s) supported by the data and/ or analysis reported in the review?** |  |  | *Data (not just citations) should be reported that support the main conclusions* |
| **10** | **Overall, how would you rate the scientific quality of this review?**   \| Extensive flaws \|  \| Major flaws \|  \| Minor flaws \|  \| Minimal flaws \| \| --- \| --- \| --- \| --- \| --- \| --- \| --- \| \| 1 \| 2 \| 3 \| 4 \| 5 \| 6 \| 7 \|   *The score for question 10, the overall scientific quality, should be based on your answers to the first nine questions. The following guidelines can be used to assist with deriving a summary score: If the "can't tell" option is used one or more times on the preceding questions, a review is likely to have minor flaws at best and it is difficult to rule out major flaws (i.e. a score of 4 or lower) .If the "no” option is used on questions 2, 4, 6 or 8, the review is likely to have major flaws (i.e. a score of 3 or less, depending on the number and degree of the flaws).* | | | **7** |

REF ID: **Bennett S. et al.** *Implementation of evidence-based, non-pharmacological interventions addressing behavior and psychological symptoms of dementia: a systematic review focused on implementation strategies* (2021)

| **Questions** | | **NO**  Can´t tell or no info | **PARTIALLY**  Incomplete report | **YES** |
| --- | --- | --- | --- | --- |
| **1** | **Were the search methods used to find evidence (primary studies) on the primary question(s) stated?** |  | *Only the search terms are presented* | *Search terms are presented, and it is also explained how these search terms are combined (by “AND”, “OR”, etc.)* |
| **2** | **Was the search for evidence reasonably comprehensive?** |  | *Only Pubmed/Medline has been searched or*  *Search period is shorter than 10 years* |  |
| **3** | **Were the criteria used for deciding which studies to include in the review reported?** |  | *Only exclusion criteria are presented* | *Inclusion criteria are explicitly described* |
| **4** | **Was bias in the selection of articles avoided?** |  | *Only a part of the selection steps was completed by two reviewers independently* | *Both the initial selection (tiltle & abstract) and full-text reading were performed by two reviewers independently* |
| **5** | **Were the criteria used for assessing the validity of the studies that were reviewed reported?** |  |  | *The reviewer(s) used explicit assessment criteria or an existing assessment checklist* |
| **6** | **Was the validity of all of the studies referred to in the text assessed using appropriate criteria (either in selecting studies for inclusion or in analyzing the studies that are cited)?** |  | *Only the rigorousness of research designs is taken into account* | *Both in the selection phase and during analysis the methodological quality of the studies is taken into account using explicit criteria* |
| **7** | **Were the methods used to combine the findings of the relevant studies (to reach a conclusion) reported?** |  |  | *A “best evidence synthesis” is performed*  *Pooling of results has taken place*  *Results are presented on the basis of a an “evidence table”* |
| **8** | **Were the findings of the relevant studies combined appropriately relative to the primary question the review addresses?** | *No attempt made to combine findings*  *No statement regarding the inappropriateness of combining findings*  *A summary (general) estimate is given* *anywhere in the abstract, the discussion or the summary section of the paper but it is not reported how that estimate was derived* |  |  |
| **9** | **Were the conclusions made by the author(s) supported by the data and/ or analysis reported in the review?** |  |  | *Data (not just citations) should be reported that support the main conclusions* |
| **10** | **Overall, how would you rate the scientific quality of this review?**   \| Extensive flaws \|  \| Major flaws \|  \| Minor flaws \|  \| Minimal flaws \| \| --- \| --- \| --- \| --- \| --- \| --- \| --- \| \| 1 \| 2 \| 3 \| 4 \| 5 \| 6 \| 7 \|   *The score for question 10, the overall scientific quality, should be based on your answers to the first nine questions. The following guidelines can be used to assist with deriving a summary score: If the "can't tell" option is used one or more times on the preceding questions, a review is likely to have minor flaws at best and it is difficult to rule out major flaws (i.e. a score of 4 or lower) .If the "no” option is used on questions 2, 4, 6 or 8, the review is likely to have major flaws (i.e. a score of 3 or less, depending on the number and degree of the flaws).* | | | **6** |

REF ID: **Noonan V.K. et al.** *Knowledge translation and implementation in spinal cord injury: a systematic review* (2014)

| **Questions** | | **NO**  Can´t tell or no info | **PARTIALLY**  Incomplete report | **YES** |
| --- | --- | --- | --- | --- |
| **1** | **Were the search methods used to find evidence (primary studies) on the primary question(s) stated?** |  | *Only the search terms are presented* | *Search terms are presented, and it is also explained how these search terms are combined (by “AND”, “OR”, etc.)* |
| **2** | **Was the search for evidence reasonably comprehensive?** |  | *Only Pubmed/Medline has been searched*  *or*  *Search period is shorter than 10 years* |  |
| **3** | **Were the criteria used for deciding which studies to include in the review reported?** |  | *Only exclusion criteria are presented* | *Inclusion criteria are explicitly described* |
| **4** | **Was bias in the selection of articles avoided?** |  | *Only a part of the selection steps was completed by two reviewers independently* | *Both the initial selection (tiltle & abstract) and full-text reading were performed by two reviewers independently* |
| **5** | **Were the criteria used for assessing the validity of the studies that were reviewed reported?** |  |  | *The reviewer(s) used explicit assessment criteria or an existing assessment checklist* |
| **6** | **Was the validity of all of the studies referred to in the text assessed using appropriate criteria (either in selecting studies for inclusion or in analyzing the studies that are cited)?** |  | *Only the rigorousness of research designs is taken into account* | *Both in the selection phase and during analysis the methodological quality of the studies is taken into account using explicit criteria* |
| **7** | **Were the methods used to combine the findings of the relevant studies (to reach a conclusion) reported?** |  |  | *A “best evidence synthesis” is performed*  *Pooling of results has taken place*  *Results are presented on the basis of a an “evidence table”* |
| **8** | **Were the findings of the relevant studies combined appropriately relative to the primary question the review addresses?** | *No attempt made to combine findings*  *No statement regarding the inappropriateness of combining findings*  *A summary (general) estimate is given* *anywhere in the abstract, the discussion or the summary section of the paper but it is not reported how that estimate was derived* |  |  |
| **9** | **Were the conclusions made by the author(s) supported by the data and/ or analysis reported in the review?** |  |  | *Data (not just citations) should be reported that support the main conclusions* |
| **10** | **Overall, how would you rate the scientific quality of this review?**   \| Extensive flaws \|  \| Major flaws \|  \| Minor flaws \|  \| Minimal flaws \| \| --- \| --- \| --- \| --- \| --- \| --- \| --- \| \| 1 \| 2 \| 3 \| 4 \| 5 \| 6 \| 7 \|   *The score for question 10, the overall scientific quality, should be based on your answers to the first nine questions. The following guidelines can be used to assist with deriving a summary score: If the "can't tell" option is used one or more times on the preceding questions, a review is likely to have minor flaws at best and it is difficult to rule out major flaws (i.e. a score of 4 or lower) .If the "no” option is used on questions 2, 4, 6 or 8, the review is likely to have major flaws (i.e. a score of 3 or less, depending on the number and degree of the flaws).* | | | **7** |

REF ID: **Yost J. et al.** *The effectiveness of knowledge translation interventions for promoting evidence-informed decision-making among nurses in tertiary care: a systematic review and meta-analysis* (2015)

| **Questions** | | **NO**  Can´t tell or no info | **PARTIALLY**  Incomplete report | **YES** |
| --- | --- | --- | --- | --- |
| **1** | **Were the search methods used to find evidence (primary studies) on the primary question(s) stated?** |  | *Only the search terms are presented* | *Search terms are presented, and it is also explained how these search terms are combined (by “AND”, “OR”, etc.)* |
| **2** | **Was the search for evidence reasonably comprehensive?** |  | *Only Pubmed/Medline has been searched*  *or*  *Search period is shorter than 10 years* |  |
| **3** | **Were the criteria used for deciding which studies to include in the review reported?** |  | *Only exclusion criteria are presented* | *Inclusion criteria are explicitly described* |
| **4** | **Was bias in the selection of articles avoided?** |  | *Only a part of the selection steps was completed by two reviewers independently* | *Both the initial selection (tiltle & abstract) and full-text reading were performed by two reviewers independently* |
| **5** | **Were the criteria used for assessing the validity of the studies that were reviewed reported?** |  |  | *The reviewer(s) used explicit assessment criteria or an existing assessment checklist* |
| **6** | **Was the validity of all of the studies referred to in the text assessed using appropriate criteria (either in selecting studies for inclusion or in analyzing the studies that are cited)?** |  | *Only the rigorousness of research designs is taken into account* | *Both in the selection phase and during analysis the methodological quality of the studies is taken into account using explicit criteria* |
| **7** | **Were the methods used to combine the findings of the relevant studies (to reach a conclusion) reported?** |  |  | *A “best evidence synthesis” is performed*  *Pooling of results has taken place*  *Results are presented on the basis of a an “evidence table”* |
| **8** | **Were the findings of the relevant studies combined appropriately relative to the primary question the review addresses?** | *No attempt made to combine findings*  *No statement regarding the inappropriateness of combining findings*  *A summary (general) estimate is given* *anywhere in the abstract, the discussion or the summary section of the paper but it is not reported how that estimate was derived* |  |  |
| **9** | **Were the conclusions made by the author(s) supported by the data and/ or analysis reported in the review?** |  |  | *Data (not just citations) should be reported that support the main conclusions* |
| **10** | **Overall, how would you rate the scientific quality of this review?**   \| Extensive flaws \|  \| Major flaws \|  \| Minor flaws \|  \| Minimal flaws \| \| --- \| --- \| --- \| --- \| --- \| --- \| --- \| \| 1 \| 2 \| 3 \| 4 \| 5 \| 6 \| 7 \|   *The score for question 10, the overall scientific quality, should be based on your answers to the first nine questions. The following guidelines can be used to assist with deriving a summary score: If the "can't tell" option is used one or more times on the preceding questions, a review is likely to have minor flaws at best and it is difficult to rule out major flaws (i.e. a score of 4 or lower) .If the "no” option is used on questions 2, 4, 6 or 8, the review is likely to have major flaws (i.e. a score of 3 or less, depending on the number and degree of the flaws).* | | | **7** |

REF ID: **Albreacht L. et al.** *Systematic review of knowledge translation strategies to promote research uptake in child health settings* (2016)

| **Questions** | | **NO**  Can´t tell or no info | **PARTIALLY**  Incomplete report | **YES** |
| --- | --- | --- | --- | --- |
| **1** | **Were the search methods used to find evidence (primary studies) on the primary question(s) stated?** |  | *Only the search terms are presented* | *Search terms are presented, and it is also explained how these search terms are combined (by “AND”, “OR”, etc.)* |
| **2** | **Was the search for evidence reasonably comprehensive?** |  | *Only Pubmed/Medline has been searched*  *or*  *Search period is shorter than 10 years* |  |
| **3** | **Were the criteria used for deciding which studies to include in the review reported?** |  | *Only exclusion criteria are presented* | *Inclusion criteria are explicitly described* |
| **4** | **Was bias in the selection of articles avoided?** |  | *Only a part of the selection steps was completed by two reviewers independently* | *Both the initial selection (tiltle & abstract) and full-text reading were performed by two reviewers independently* |
| **5** | **Were the criteria used for assessing the validity of the studies that were reviewed reported?** |  |  | *The reviewer(s) used explicit assessment criteria or an existing assessment checklist* |
| **6** | **Was the validity of all of the studies referred to in the text assessed using appropriate criteria (either in selecting studies for inclusion or in analyzing the studies that are cited)?** |  | *Only the rigorousness of research designs is taken into account* | *Both in the selection phase and during analysis the methodological quality of the studies is taken into account using explicit criteria* |
| **7** | **Were the methods used to combine the findings of the relevant studies (to reach a conclusion) reported?** |  |  | *A “best evidence synthesis” is performed*  *Pooling of results has taken place*  *Results are presented on the basis of a an “evidence table”* |
| **8** | **Were the findings of the relevant studies combined appropriately relative to the primary question the review addresses?** | *No attempt made to combine findings*  *No statement regarding the inappropriateness of combining findings*  *A summary (general) estimate is given* *anywhere in the abstract, the discussion or the summary section of the paper but it is not reported how that estimate was derived* |  |  |
| **9** | **Were the conclusions made by the author(s) supported by the data and/ or analysis reported in the review?** |  |  | *Data (not just citations) should be reported that support the main conclusions* |
| **10** | **Overall, how would you rate the scientific quality of this review?**   \| Extensive flaws \|  \| Major flaws \|  \| Minor flaws \|  \| Minimal flaws \| \| --- \| --- \| --- \| --- \| --- \| --- \| --- \| \| 1 \| 2 \| 3 \| 4 \| 5 \| 6 \| 7 \|   *The score for question 10, the overall scientific quality, should be based on your answers to the first nine questions. The following guidelines can be used to assist with deriving a summary score: If the "can't tell" option is used one or more times on the preceding questions, a review is likely to have minor flaws at best and it is difficult to rule out major flaws (i.e. a score of 4 or lower) .If the "no” option is used on questions 2, 4, 6 or 8, the review is likely to have major flaws (i.e. a score of 3 or less, depending on the number and degree of the flaws).* | | | **7** |

REF ID: **Scott D. et al.** *Systematic review of knowledge translation strategies in the allied health professions* (2012)

| **Questions** | | **NO**  Can´t tell or no info | **PARTIALLY**  Incomplete report | **YES** |
| --- | --- | --- | --- | --- |
| **1** | **Were the search methods used to find evidence (primary studies) on the primary question(s) stated?** |  | *Only the search terms are presented* | *Search terms are presented, and it is also explained how these search terms are combined (by “AND”, “OR”, etc.)* |
| **2** | **Was the search for evidence reasonably comprehensive?** |  | *Only Pubmed/Medline has been searched*  *or*  *Search period is shorter than 10 years* |  |
| **3** | **Were the criteria used for deciding which studies to include in the review reported?** |  | *Only exclusion criteria are presented* | *Inclusion criteria are explicitly described* |
| **4** | **Was bias in the selection of articles avoided?** |  | *Only a part of the selection steps was completed by two reviewers independently* | *Both the initial selection (tiltle & abstract) and full-text reading were performed by two reviewers independently* |
| **5** | **Were the criteria used for assessing the validity of the studies that were reviewed reported?** |  |  | *The reviewer(s) used explicit assessment criteria or an existing assessment checklist* |
| **6** | **Was the validity of all of the studies referred to in the text assessed using appropriate criteria (either in selecting studies for inclusion or in analyzing the studies that are cited)?** |  | *Only the rigorousness of research designs is taken into account* | *Both in the selection phase and during analysis the methodological quality of the studies is taken into account using explicit criteria* |
| **7** | **Were the methods used to combine the findings of the relevant studies (to reach a conclusion) reported?** |  |  | *A “best evidence synthesis” is performed*  *Pooling of results has taken place*  *Results are presented on the basis of a an “evidence table”* |
| **8** | **Were the findings of the relevant studies combined appropriately relative to the primary question the review addresses?** | *No attempt made to combine findings*  *No statement regarding the inappropriateness of combining findings*  *A summary (general) estimate is given* *anywhere in the abstract, the discussion or the summary section of the paper but it is not reported how that estimate was derived* |  |  |
| **9** | **Were the conclusions made by the author(s) supported by the data and/ or analysis reported in the review?** |  |  | *Data (not just citations) should be reported that support the main conclusions* |
| **10** | **Overall, how would you rate the scientific quality of this review?**   \| Extensive flaws \|  \| Major flaws \|  \| Minor flaws \|  \| Minimal flaws \| \| --- \| --- \| --- \| --- \| --- \| --- \| --- \| \| 1 \| 2 \| 3 \| 4 \| 5 \| 6 \| 7 \|   *The score for question 10, the overall scientific quality, should be based on your answers to the first nine questions. The following guidelines can be used to assist with deriving a summary score: If the "can't tell" option is used one or more times on the preceding questions, a review is likely to have minor flaws at best and it is difficult to rule out major flaws (i.e. a score of 4 or lower) .If the "no” option is used on questions 2, 4, 6 or 8, the review is likely to have major flaws (i.e. a score of 3 or less, depending on the number and degree of the flaws).* | | | **7** |

REF ID: **Campbell A. et al.** *Knowledge translation strategies used by healthcare professionals in child health settings: an updated systematic review* (2019)

| **Questions** | | **NO**  Can´t tell or no info | **PARTIALLY**  Incomplete report | **YES** |
| --- | --- | --- | --- | --- |
| **1** | **Were the search methods used to find evidence (primary studies) on the primary question(s) stated?** |  | *Only the search terms are presented* | *Search terms are presented, and it is also explained how these search terms are combined (by “AND”, “OR”, etc.)* |
| **2** | **Was the search for evidence reasonably comprehensive?** |  | *Only Pubmed/Medline has been searched*  *or*  *Search period is shorter than 10 years* |  |
| **3** | **Were the criteria used for deciding which studies to include in the review reported?** |  | *Only exclusion criteria are presented* | *Inclusion criteria are explicitly described* |
| **4** | **Was bias in the selection of articles avoided?** |  | *Only a part of the selection steps was completed by two reviewers independently* | *Both the initial selection (tiltle & abstract) and full-text reading were performed by two reviewers independently* |
| **5** | **Were the criteria used for assessing the validity of the studies that were reviewed reported?** |  |  | *The reviewer(s) used explicit assessment criteria or an existing assessment checklist* |
| **6** | **Was the validity of all of the studies referred to in the text assessed using appropriate criteria (either in selecting studies for inclusion or in analyzing the studies that are cited)?** |  | *Only the rigorousness of research designs is taken into account* | *Both in the selection phase and during analysis the methodological quality of the studies is taken into account using explicit criteria* |
| **7** | **Were the methods used to combine the findings of the relevant studies (to reach a conclusion) reported?** |  |  | *A “best evidence synthesis” is performed*  *Pooling of results has taken place*  *Results are presented on the basis of a an “evidence table”* |
| **8** | **Were the findings of the relevant studies combined appropriately relative to the primary question the review addresses?** | *No attempt made to combine findings*  *No statement regarding the inappropriateness of combining findings*  *A summary (general) estimate is given* *anywhere in the abstract, the discussion or the summary section of the paper but it is not reported how that estimate was derived* |  |  |
| **9** | **Were the conclusions made by the author(s) supported by the data and/ or analysis reported in the review?** |  |  | *Data (not just citations) should be reported that support the main conclusions* |
| **10** | **Overall, how would you rate the scientific quality of this review?**   \| Extensive flaws \|  \| Major flaws \|  \| Minor flaws \|  \| Minimal flaws \| \| --- \| --- \| --- \| --- \| --- \| --- \| --- \| \| 1 \| 2 \| 3 \| 4 \| 5 \| 6 \| 7 \|   *The score for question 10, the overall scientific quality, should be based on your answers to the first nine questions. The following guidelines can be used to assist with deriving a summary score: If the "can't tell" option is used one or more times on the preceding questions, a review is likely to have minor flaws at best and it is difficult to rule out major flaws (i.e. a score of 4 or lower) .If the "no” option is used on questions 2, 4, 6 or 8, the review is likely to have major flaws (i.e. a score of 3 or less, depending on the number and degree of the flaws).* | | | **7** |

REF ID: **Flodgren G. et al.** *Local opinion leaders: effects on professional practice and healthcare outcomes* (2019)

| **Questions** | | **NO**  Can´t tell or no info | **PARTIALLY**  Incomplete report | **YES** |
| --- | --- | --- | --- | --- |
| **1** | **Were the search methods used to find evidence (primary studies) on the primary question(s) stated?** |  | *Only the search terms are presented* | *Search terms are presented, and it is also explained how these search terms are combined (by “AND”, “OR”, etc.)* |
| **2** | **Was the search for evidence reasonably comprehensive?** |  | *Only Pubmed/Medline has been searched*  *or*  *Search period is shorter than 10 years* | *Shorter than 10 years but it was an update on a previous review* |
| **3** | **Were the criteria used for deciding which studies to include in the review reported?** |  | *Only exclusion criteria are presented* | *Inclusion criteria are explicitly described* |
| **4** | **Was bias in the selection of articles avoided?** |  | *Only a part of the selection steps was completed by two reviewers independently* | *Both the initial selection (tiltle & abstract) and full-text reading were performed by two reviewers independently* |
| **5** | **Were the criteria used for assessing the validity of the studies that were reviewed reported?** |  |  | *The reviewer(s) used explicit assessment criteria or an existing assessment checklist* |
| **6** | **Was the validity of all of the studies referred to in the text assessed using appropriate criteria (either in selecting studies for inclusion or in analyzing the studies that are cited)?** |  | *Only the rigorousness of research designs is taken into account* | *Both in the selection phase and during analysis the methodological quality of the studies is taken into account using explicit criteria* |
| **7** | **Were the methods used to combine the findings of the relevant studies (to reach a conclusion) reported?** |  |  | *A “best evidence synthesis” is performed*  *Pooling of results has taken place*  *Results are presented on the basis of a an “evidence table”* |
| **8** | **Were the findings of the relevant studies combined appropriately relative to the primary question the review addresses?** | *No attempt made to combine findings*  *No statement regarding the inappropriateness of combining findings*  *A summary (general) estimate is given* *anywhere in the abstract, the discussion or the summary section of the paper but it is not reported how that estimate was derived* |  |  |
| **9** | **Were the conclusions made by the author(s) supported by the data and/ or analysis reported in the review?** |  |  | *Data (not just citations) should be reported that support the main conclusions* |
| **10** | **Overall, how would you rate the scientific quality of this review?**   \| Extensive flaws \|  \| Major flaws \|  \| Minor flaws \|  \| Minimal flaws \| \| --- \| --- \| --- \| --- \| --- \| --- \| --- \| \| 1 \| 2 \| 3 \| 4 \| 5 \| 6 \| 7 \|   *The score for question 10, the overall scientific quality, should be based on your answers to the first nine questions. The following guidelines can be used to assist with deriving a summary score: If the "can't tell" option is used one or more times on the preceding questions, a review is likely to have minor flaws at best and it is difficult to rule out major flaws (i.e. a score of 4 or lower) .If the "no” option is used on questions 2, 4, 6 or 8, the review is likely to have major flaws (i.e. a score of 3 or less, depending on the number and degree of the flaws).* | | | **7** |

REF ID: **Pantoja T. et al.** *Manually-generated reminders delivered on paper: effects on professional practice and patient outcomes* (2019)

| **Questions** | | **NO**  Can´t tell or no info | **PARTIALLY**  Incomplete report | **YES** |
| --- | --- | --- | --- | --- |
| **1** | **Were the search methods used to find evidence (primary studies) on the primary question(s) stated?** |  | *Only the search terms are presented* | *Search terms are presented, and it is also explained how these search terms are combined (by “AND”, “OR”, etc.)* |
| **2** | **Was the search for evidence reasonably comprehensive?** |  | *Only Pubmed/Medline has been searched*  *or*  *Search period is shorter than 10 years* |  |
| **3** | **Were the criteria used for deciding which studies to include in the review reported?** |  | *Only exclusion criteria are presented* | *Inclusion criteria are explicitly described* |
| **4** | **Was bias in the selection of articles avoided?** |  | *Only a part of the selection steps was completed by two reviewers independently* | *Both the initial selection (tiltle & abstract) and full-text reading were performed by two reviewers independently* |
| **5** | **Were the criteria used for assessing the validity of the studies that were reviewed reported?** |  |  | *The reviewer(s) used explicit assessment criteria or an existing assessment checklist* |
| **6** | **Was the validity of all of the studies referred to in the text assessed using appropriate criteria (either in selecting studies for inclusion or in analyzing the studies that are cited)?** |  | *Only the rigorousness of research designs is taken into account* | *Both in the selection phase and during analysis the methodological quality of the studies is taken into account using explicit criteria* |
| **7** | **Were the methods used to combine the findings of the relevant studies (to reach a conclusion) reported?** |  |  | *A “best evidence synthesis” is performed*  *Pooling of results has taken place*  *Results are presented on the basis of a an “evidence table”* |
| **8** | **Were the findings of the relevant studies combined appropriately relative to the primary question the review addresses?** | *No attempt made to combine findings*  *No statement regarding the inappropriateness of combining findings*  *A summary (general) estimate is given* *anywhere in the abstract, the discussion or the summary section of the paper but it is not reported how that estimate was derived* |  |  |
| **9** | **Were the conclusions made by the author(s) supported by the data and/ or analysis reported in the review?** |  |  | *Data (not just citations) should be reported that support the main conclusions* |
| **10** | **Overall, how would you rate the scientific quality of this review?**   \| Extensive flaws \|  \| Major flaws \|  \| Minor flaws \|  \| Minimal flaws \| \| --- \| --- \| --- \| --- \| --- \| --- \| --- \| \| 1 \| 2 \| 3 \| 4 \| 5 \| 6 \| 7 \|   *The score for question 10, the overall scientific quality, should be based on your answers to the first nine questions. The following guidelines can be used to assist with deriving a summary score: If the "can't tell" option is used one or more times on the preceding questions, a review is likely to have minor flaws at best and it is difficult to rule out major flaws (i.e. a score of 4 or lower) .If the "no” option is used on questions 2, 4, 6 or 8, the review is likely to have major flaws (i.e. a score of 3 or less, depending on the number and degree of the flaws).* | | | **7** |

REF ID: **Bird M.L. et al.** *Moving stroke rehabilitation evidence into practice: a systematic review of randomized controlled trials* (2019)

| **Questions** | | **NO**  Can´t tell or no info | **PARTIALLY**  Incomplete report | **YES** |
| --- | --- | --- | --- | --- |
| **1** | **Were the search methods used to find evidence (primary studies) on the primary question(s) stated?** |  | *Only the search terms are presented* | *Search terms are presented, and it is also explained how these search terms are combined (by “AND”, “OR”, etc.)* |
| **2** | **Was the search for evidence reasonably comprehensive?** |  | *Only Pubmed/Medline has been searched*  *or*  *Search period is shorter than 10 years* |  |
| **3** | **Were the criteria used for deciding which studies to include in the review reported?** |  | *Only exclusion criteria are presented* | *Inclusion criteria are explicitly described* |
| **4** | **Was bias in the selection of articles avoided?** |  | *Only a part of the selection steps was completed by two reviewers independently* | *Both the initial selection (tiltle & abstract) and full-text reading were performed by two reviewers independently* |
| **5** | **Were the criteria used for assessing the validity of the studies that were reviewed reported?** |  |  | *The reviewer(s) used explicit assessment criteria or an existing assessment checklist* |
| **6** | **Was the validity of all of the studies referred to in the text assessed using appropriate criteria (either in selecting studies for inclusion or in analyzing the studies that are cited)?** |  | *Only the rigorousness of research designs is taken into account* | *Both in the selection phase and during analysis the methodological quality of the studies is taken into account using explicit criteria* |
| **7** | **Were the methods used to combine the findings of the relevant studies (to reach a conclusion) reported?** |  |  | *A “best evidence synthesis” is performed*  *Pooling of results has taken place*  *Results are presented on the basis of a an “evidence table”* |
| **8** | **Were the findings of the relevant studies combined appropriately relative to the primary question the review addresses?** | *No attempt made to combine findings*  *No statement regarding the inappropriateness of combining findings*  *A summary (general) estimate is given* *anywhere in the abstract, the discussion or the summary section of the paper but it is not reported how that estimate was derived* |  |  |
| **9** | **Were the conclusions made by the author(s) supported by the data and/ or analysis reported in the review?** |  |  | *Data (not just citations) should be reported that support the main conclusions* |
| **10** | **Overall, how would you rate the scientific quality of this review?**   \| Extensive flaws \|  \| Major flaws \|  \| Minor flaws \|  \| Minimal flaws \| \| --- \| --- \| --- \| --- \| --- \| --- \| --- \| \| 1 \| 2 \| 3 \| 4 \| 5 \| 6 \| 7 \|   *The score for question 10, the overall scientific quality, should be based on your answers to the first nine questions. The following guidelines can be used to assist with deriving a summary score: If the "can't tell" option is used one or more times on the preceding questions, a review is likely to have minor flaws at best and it is difficult to rule out major flaws (i.e. a score of 4 or lower) .If the "no” option is used on questions 2, 4, 6 or 8, the review is likely to have major flaws (i.e. a score of 3 or less, depending on the number and degree of the flaws).* | | | **7** |

REF ID: **Goorts K., Dizon J. & Milanese S.** *The effectiveness of implementation strategies for promoting evidence informed interventions in allied healthcare: a systematic review* (2021)

| **Questions** | | **NO**  Can´t tell or no info | **PARTIALLY**  Incomplete report | **YES** |
| --- | --- | --- | --- | --- |
| **1** | **Were the search methods used to find evidence (primary studies) on the primary question(s) stated?** |  | *Only the search terms are presented* | *Search terms are presented, and it is also explained how these search terms are combined (by “AND”, “OR”, etc.)* |
| **2** | **Was the search for evidence reasonably comprehensive?** |  | *Only Pubmed/Medline has been searched*  *or*  *Search period is shorter than 10 years* |  |
| **3** | **Were the criteria used for deciding which studies to include in the review reported?** |  | *Only exclusion criteria are presented* | *Inclusion criteria are explicitly described* |
| **4** | **Was bias in the selection of articles avoided?** |  | *Only a part of the selection steps was completed by two reviewers independently* | *Both the initial selection (tiltle & abstract) and full-text reading were performed by two reviewers independently* |
| **5** | **Were the criteria used for assessing the validity of the studies that were reviewed reported?** |  |  | *The reviewer(s) used explicit assessment criteria or an existing assessment checklist* |
| **6** | **Was the validity of all of the studies referred to in the text assessed using appropriate criteria (either in selecting studies for inclusion or in analyzing the studies that are cited)?** |  | *Only the rigorousness of research designs is taken into account* | *Both in the selection phase and during analysis the methodological quality of the studies is taken into account using explicit criteria* |
| **7** | **Were the methods used to combine the findings of the relevant studies (to reach a conclusion) reported?** |  |  | *A “best evidence synthesis” is performed*  *Pooling of results has taken place*  *Results are presented on the basis of a an “evidence table”* |
| **8** | **Were the findings of the relevant studies combined appropriately relative to the primary question the review addresses?** | *No attempt made to combine findings*  *No statement regarding the inappropriateness of combining findings*  *A summary (general) estimate is given* *anywhere in the abstract, the discussion or the summary section of the paper but it is not reported how that estimate was derived* |  |  |
| **9** | **Were the conclusions made by the author(s) supported by the data and/ or analysis reported in the review?** |  |  | *Data (not just citations) should be reported that support the main conclusions* |
| **10** | **Overall, how would you rate the scientific quality of this review?**   \| Extensive flaws \|  \| Major flaws \|  \| Minor flaws \|  \| Minimal flaws \| \| --- \| --- \| --- \| --- \| --- \| --- \| --- \| \| 1 \| 2 \| 3 \| 4 \| 5 \| 6 \| 7 \|   *The score for question 10, the overall scientific quality, should be based on your answers to the first nine questions. The following guidelines can be used to assist with deriving a summary score: If the "can't tell" option is used one or more times on the preceding questions, a review is likely to have minor flaws at best and it is difficult to rule out major flaws (i.e. a score of 4 or lower) .If the "no” option is used on questions 2, 4, 6 or 8, the review is likely to have major flaws (i.e. a score of 3 or less, depending on the number and degree of the flaws).* | | | **7** |

REF ID: **Zadro J.R. et al.** *Effectiveness of implementation strategies to improve adherence of physical therapist treatment choices to clinical practice guidelines for musculoskeletal conditions: systematic review* (2020)

| **Questions** | | **NO**  Can´t tell or no info | **PARTIALLY**  Incomplete report | **YES** |
| --- | --- | --- | --- | --- |
| **1** | **Were the search methods used to find evidence (primary studies) on the primary question(s) stated?** |  | *Only the search terms are presented* | *Search terms are presented, and it is also explained how these search terms are combined (by “AND”, “OR”, etc.)* |
| **2** | **Was the search for evidence reasonably comprehensive?** |  | *Only Pubmed/Medline has been searched*  *or*  *Search period is shorter than 10 years* |  |
| **3** | **Were the criteria used for deciding which studies to include in the review reported?** |  | *Only exclusion criteria are presented* | *Inclusion criteria are explicitly described* |
| **4** | **Was bias in the selection of articles avoided?** |  | *Only a part of the selection steps was completed by two reviewers independently* | *Both the initial selection (tiltle & abstract) and full-text reading were performed by two reviewers independently* |
| **5** | **Were the criteria used for assessing the validity of the studies that were reviewed reported?** |  |  | *The reviewer(s) used explicit assessment criteria or an existing assessment checklist* |
| **6** | **Was the validity of all of the studies referred to in the text assessed using appropriate criteria (either in selecting studies for inclusion or in analyzing the studies that are cited)?** |  | *Only the rigorousness of research designs is taken into account* | *Both in the selection phase and during analysis the methodological quality of the studies is taken into account using explicit criteria* |
| **7** | **Were the methods used to combine the findings of the relevant studies (to reach a conclusion) reported?** |  |  | *A “best evidence synthesis” is performed*  *Pooling of results has taken place*  *Results are presented on the basis of a an “evidence table”* |
| **8** | **Were the findings of the relevant studies combined appropriately relative to the primary question the review addresses?** | *No attempt made to combine findings*  *No statement regarding the inappropriateness of combining findings*  *A summary (general) estimate is given* *anywhere in the abstract, the discussion or the summary section of the paper but it is not reported how that estimate was derived* |  |  |
| **9** | **Were the conclusions made by the author(s) supported by the data and/ or analysis reported in the review?** |  |  | *Data (not just citations) should be reported that support the main conclusions* |
| **10** | **Overall, how would you rate the scientific quality of this review?**   \| Extensive flaws \|  \| Major flaws \|  \| Minor flaws \|  \| Minimal flaws \| \| --- \| --- \| --- \| --- \| --- \| --- \| --- \| \| 1 \| 2 \| 3 \| 4 \| 5 \| 6 \| 7 \|   *The score for question 10, the overall scientific quality, should be based on your answers to the first nine questions. The following guidelines can be used to assist with deriving a summary score: If the "can't tell" option is used one or more times on the preceding questions, a review is likely to have minor flaws at best and it is difficult to rule out major flaws (i.e. a score of 4 or lower) .If the "no” option is used on questions 2, 4, 6 or 8, the review is likely to have major flaws (i.e. a score of 3 or less, depending on the number and degree of the flaws).* | | | **7** |

REF ID: **Menon A. et al.** *Strategies for rehabilitation professionals to move evidence-based knowledge into practice: a systematic review* (2009)

| **Questions** | | **NO**  Can´t tell or no info | **PARTIALLY**  Incomplete report | **YES** |
| --- | --- | --- | --- | --- |
| **1** | **Were the search methods used to find evidence (primary studies) on the primary question(s) stated?** |  | *Only the search terms are presented* | *Search terms are presented, and it is also explained how these search terms are combined (by “AND”, “OR”, etc.)* |
| **2** | **Was the search for evidence reasonably comprehensive?** |  | *Only Pubmed/Medline has been searched or*  *Search period is shorter than 10 years* |  |
| **3** | **Were the criteria used for deciding which studies to include in the review reported?** |  | *Only exclusion criteria are presented* | *Inclusion criteria are explicitly described* |
| **4** | **Was bias in the selection of articles avoided?** |  | *Only a part of the selection steps was completed by two reviewers independently* | *Both the initial selection (tiltle & abstract) and full-text reading were performed by two reviewers independently* |
| **5** | **Were the criteria used for assessing the validity of the studies that were reviewed reported?** |  |  | *The reviewer(s) used explicit assessment criteria or an existing assessment checklist* |
| **6** | **Was the validity of all of the studies referred to in the text assessed using appropriate criteria (either in selecting studies for inclusion or in analyzing the studies that are cited)?** |  | *Only the rigorousness of research designs is taken into account* | *Both in the selection phase and during analysis the methodological quality of the studies is taken into account using explicit criteria* |
| **7** | **Were the methods used to combine the findings of the relevant studies (to reach a conclusion) reported?** |  |  | *A “best evidence synthesis” is performed*  *Pooling of results has taken place*  *Results are presented on the basis of a an “evidence table”* |
| **8** | **Were the findings of the relevant studies combined appropriately relative to the primary question the review addresses?** | *No attempt made to combine findings*  *No statement regarding the inappropriateness of combining findings*  *A summary (general) estimate is given* *anywhere in the abstract, the discussion or the summary section of the paper but it is not reported how that estimate was derived* |  |  |
| **9** | **Were the conclusions made by the author(s) supported by the data and/ or analysis reported in the review?** |  |  | *Data (not just citations) should be reported that support the main conclusions* |
| **10** | **Overall, how would you rate the scientific quality of this review?**   \| Extensive flaws \|  \| Major flaws \|  \| Minor flaws \|  \| Minimal flaws \| \| --- \| --- \| --- \| --- \| --- \| --- \| --- \| \| 1 \| 2 \| 3 \| 4 \| 5 \| 6 \| 7 \|   *The score for question 10, the overall scientific quality, should be based on your answers to the first nine questions. The following guidelines can be used to assist with deriving a summary score: If the "can't tell" option is used one or more times on the preceding questions, a review is likely to have minor flaws at best and it is difficult to rule out major flaws (i.e. a score of 4 or lower) .If the "no” option is used on questions 2, 4, 6 or 8, the review is likely to have major flaws (i.e. a score of 3 or less, depending on the number and degree of the flaws).* | | | **7** |

REF ID: **Jamal A., McKenzie K. & Clark M.** *The impact of health information technology on the quality of medical and health care: a systematic review* (2009)

| **Questions** | | **NO**  Can´t tell or no info | **PARTIALLY**  Incomplete report | **YES** |
| --- | --- | --- | --- | --- |
| **1** | **Were the search methods used to find evidence (primary studies) on the primary question(s) stated?** |  | *Only the search terms are presented* | *Search terms are presented, and it is also explained how these search terms are combined (by “AND”, “OR”, etc.)* |
| **2** | **Was the search for evidence reasonably comprehensive?** |  | *Only Pubmed/Medline has been searched or*  *Search period is shorter than 10 years* |  |
| **3** | **Were the criteria used for deciding which studies to include in the review reported?** |  | *Only exclusion criteria are presented* | *Inclusion criteria are explicitly described* |
| **4** | **Was bias in the selection of articles avoided?** |  | *Only a part of the selection steps was completed by two reviewers independently* | *Both the initial selection (tiltle & abstract) and full-text reading were performed by two reviewers independently* |
| **5** | **Were the criteria used for assessing the validity of the studies that were reviewed reported?** |  |  | *The reviewer(s) used explicit assessment criteria or an existing assessment checklist* |
| **6** | **Was the validity of all of the studies referred to in the text assessed using appropriate criteria (either in selecting studies for inclusion or in analyzing the studies that are cited)?** |  | *Only the rigorousness of research designs is taken into account* | *Both in the selection phase and during analysis the methodological quality of the studies is taken into account using explicit criteria* |
| **7** | **Were the methods used to combine the findings of the relevant studies (to reach a conclusion) reported?** |  |  | *A “best evidence synthesis” is performed*  *Pooling of results has taken place*  *Results are presented on the basis of a an “evidence table”* |
| **8** | **Were the findings of the relevant studies combined appropriately relative to the primary question the review addresses?** | *No attempt made to combine findings*  *No statement regarding the inappropriateness of combining findings*  *A summary (general) estimate is given* *anywhere in the abstract, the discussion or the summary section of the paper but it is not reported how that estimate was derived* |  |  |
| **9** | **Were the conclusions made by the author(s) supported by the data and/ or analysis reported in the review?** |  |  | *Data (not just citations) should be reported that support the main conclusions* |
| **10** | **Overall, how would you rate the scientific quality of this review?**   \| Extensive flaws \|  \| Major flaws \|  \| Minor flaws \|  \| Minimal flaws \| \| --- \| --- \| --- \| --- \| --- \| --- \| --- \| \| 1 \| 2 \| 3 \| 4 \| 5 \| 6 \| 7 \|   *The score for question 10, the overall scientific quality, should be based on your answers to the first nine questions. The following guidelines can be used to assist with deriving a summary score: If the "can't tell" option is used one or more times on the preceding questions, a review is likely to have minor flaws at best and it is difficult to rule out major flaws (i.e. a score of 4 or lower) .If the "no” option is used on questions 2, 4, 6 or 8, the review is likely to have major flaws (i.e. a score of 3 or less, depending on the number and degree of the flaws).* | | | **3** |

REF ID: **Jones C.A. et al.** *Translating knowledge in rehabilitation: systematic review* (2015)

| **Questions** | | **NO**  Can´t tell or no info | **PARTIALLY**  Incomplete report | **YES** |
| --- | --- | --- | --- | --- |
| **1** | **Were the search methods used to find evidence (primary studies) on the primary question(s) stated?** |  | *Only the search terms are presented* | *Search terms are presented, and it is also explained how these search terms are combined (by “AND”, “OR”, etc.)* |
| **2** | **Was the search for evidence reasonably comprehensive?** |  | *Only Pubmed/Medline has been searched*  *or*  *Search period is shorter than 10 years* |  |
| **3** | **Were the criteria used for deciding which studies to include in the review reported?** |  | *Only exclusion criteria are presented* | *Inclusion criteria are explicitly described* |
| **4** | **Was bias in the selection of articles avoided?** |  | *Only a part of the selection steps was completed by two reviewers independently* | *Both the initial selection (tiltle & abstract) and full-text reading were performed by two reviewers independently* |
| **5** | **Were the criteria used for assessing the validity of the studies that were reviewed reported?** |  |  | *The reviewer(s) used explicit assessment criteria or an existing assessment checklist* |
| **6** | **Was the validity of all of the studies referred to in the text assessed using appropriate criteria (either in selecting studies for inclusion or in analyzing the studies that are cited)?** |  | *Only the rigorousness of research designs is taken into account* | *Both in the selection phase and during analysis the methodological quality of the studies is taken into account using explicit criteria* |
| **7** | **Were the methods used to combine the findings of the relevant studies (to reach a conclusion) reported?** |  |  | *A “best evidence synthesis” is performed*  *Pooling of results has taken place*  *Results are presented on the basis of a an “evidence table”* |
| **8** | **Were the findings of the relevant studies combined appropriately relative to the primary question the review addresses?** | *No attempt made to combine findings*  *No statement regarding the inappropriateness of combining findings*  *A summary (general) estimate is given* *anywhere in the abstract, the discussion or the summary section of the paper but it is not reported how that estimate was derived* |  |  |
| **9** | **Were the conclusions made by the author(s) supported by the data and/ or analysis reported in the review?** |  |  | *Data (not just citations) should be reported that support the main conclusions* |
| **10** | **Overall, how would you rate the scientific quality of this review?**   \| Extensive flaws \|  \| Major flaws \|  \| Minor flaws \|  \| Minimal flaws \| \| --- \| --- \| --- \| --- \| --- \| --- \| --- \| \| 1 \| 2 \| 3 \| 4 \| 5 \| 6 \| 7 \|   *The score for question 10, the overall scientific quality, should be based on your answers to the first nine questions. The following guidelines can be used to assist with deriving a summary score: If the "can't tell" option is used one or more times on the preceding questions, a review is likely to have minor flaws at best and it is difficult to rule out major flaws (i.e. a score of 4 or lower) .If the "no” option is used on questions 2, 4, 6 or 8, the review is likely to have major flaws (i.e. a score of 3 or less, depending on the number and degree of the flaws).* | | | **7** |

REF ID: **Van Der Veer S.N. et al.** *Translating knowledge on best practice into improving quality of RRT care: A systematic review of implementation strategies* (2011)

| **Questions** | | **NO**  Can´t tell or no info | **PARTIALLY**  Incomplete report | **YES** |
| --- | --- | --- | --- | --- |
| **1** | **Were the search methods used to find evidence (primary studies) on the primary question(s) stated?** |  | *Only the search terms are presented* | *Search terms are presented, and it is also explained how these search terms are combined (by “AND”, “OR”, etc.)* |
| **2** | **Was the search for evidence reasonably comprehensive?** |  | *Only Pubmed/Medline has been searched*  *or*  *Search period is shorter than 10 years* |  |
| **3** | **Were the criteria used for deciding which studies to include in the review reported?** |  | *Only exclusion criteria are presented* | *Inclusion criteria are explicitly described* |
| **4** | **Was bias in the selection of articles avoided?** |  | *Only a part of the selection steps was completed by two reviewers independently* | *Both the initial selection (tiltle & abstract) and full-text reading were performed by two reviewers independently* |
| **5** | **Were the criteria used for assessing the validity of the studies that were reviewed reported?** |  |  | *The reviewer(s) used explicit assessment criteria or an existing assessment checklist* |
| **6** | **Was the validity of all of the studies referred to in the text assessed using appropriate criteria (either in selecting studies for inclusion or in analyzing the studies that are cited)?** |  | *Only the rigorousness of research designs is taken into account* | *Both in the selection phase and during analysis the methodological quality of the studies is taken into account using explicit criteria* |
| **7** | **Were the methods used to combine the findings of the relevant studies (to reach a conclusion) reported?** |  |  | *A “best evidence synthesis” is performed*  *Pooling of results has taken place*  *Results are presented on the basis of a an “evidence table”* |
| **8** | **Were the findings of the relevant studies combined appropriately relative to the primary question the review addresses?** | *No attempt made to combine findings*  *No statement regarding the inappropriateness of combining findings*  *A summary (general) estimate is given* *anywhere in the abstract, the discussion or the summary section of the paper but it is not reported how that estimate was derived* |  |  |
| **9** | **Were the conclusions made by the author(s) supported by the data and/ or analysis reported in the review?** |  |  | *Data (not just citations) should be reported that support the main conclusions* |
| **10** | **Overall, how would you rate the scientific quality of this review?**   \| Extensive flaws \|  \| Major flaws \|  \| Minor flaws \|  \| Minimal flaws \| \| --- \| --- \| --- \| --- \| --- \| --- \| --- \| \| 1 \| 2 \| 3 \| 4 \| 5 \| 6 \| 7 \|   *The score for question 10, the overall scientific quality, should be based on your answers to the first nine questions. The following guidelines can be used to assist with deriving a summary score: If the "can't tell" option is used one or more times on the preceding questions, a review is likely to have minor flaws at best and it is difficult to rule out major flaws (i.e. a score of 4 or lower) .If the "no” option is used on questions 2, 4, 6 or 8, the review is likely to have major flaws (i.e. a score of 3 or less, depending on the number and degree of the flaws).* | | | **6** |

REF ID: **Bhatt N.R. et al.** *A systematic review of the use of social media for dissemination of clinical practice guidelines* (2021)

| **Questions** | | **NO**  Can´t tell or no info | **PARTIALLY**  Incomplete report | **YES** |
| --- | --- | --- | --- | --- |
| **1** | **Were the search methods used to find evidence (primary studies) on the primary question(s) stated?** |  | *Only the search terms are presented* | *Search terms are presented, and it is also explained how these search terms are combined (by “AND”, “OR”, etc.)* |
| **2** | **Was the search for evidence reasonably comprehensive?** |  | *Only Pubmed/Medline has been searched*  *or*  *Search period is shorter than 10 years* |  |
| **3** | **Were the criteria used for deciding which studies to include in the review reported?** |  | *Only exclusion criteria are presented* | *Inclusion criteria are explicitly described* |
| **4** | **Was bias in the selection of articles avoided?** |  | *Only a part of the selection steps was completed by two reviewers independently* | *Both the initial selection (tiltle & abstract) and full-text reading were performed by two reviewers independently* |
| **5** | **Were the criteria used for assessing the validity of the studies that were reviewed reported?** |  |  | *The reviewer(s) used explicit assessment criteria or an existing assessment checklist* |
| **6** | **Was the validity of all of the studies referred to in the text assessed using appropriate criteria (either in selecting studies for inclusion or in analyzing the studies that are cited)?** |  | *Only the rigorousness of research designs is taken into account* | *Both in the selection phase and during analysis the methodological quality of the studies is taken into account using explicit criteria* |
| **7** | **Were the methods used to combine the findings of the relevant studies (to reach a conclusion) reported?** |  |  | *A “best evidence synthesis” is performed*  *Pooling of results has taken place*  *Results are presented on the basis of a an “evidence table”* |
| **8** | **Were the findings of the relevant studies combined appropriately relative to the primary question the review addresses?** | *No attempt made to combine findings*  *No statement regarding the inappropriateness of combining findings*  *A summary (general) estimate is given* *anywhere in the abstract, the discussion or the summary section of the paper but it is not reported how that estimate was derived* |  |  |
| **9** | **Were the conclusions made by the author(s) supported by the data and/ or analysis reported in the review?** |  |  | *Data (not just citations) should be reported that support the main conclusions* |
| **10** | **Overall, how would you rate the scientific quality of this review?**   \| Extensive flaws \|  \| Major flaws \|  \| Minor flaws \|  \| Minimal flaws \| \| --- \| --- \| --- \| --- \| --- \| --- \| --- \| \| 1 \| 2 \| 3 \| 4 \| 5 \| 6 \| 7 \|   *The score for question 10, the overall scientific quality, should be based on your answers to the first nine questions. The following guidelines can be used to assist with deriving a summary score: If the "can't tell" option is used one or more times on the preceding questions, a review is likely to have minor flaws at best and it is difficult to rule out major flaws (i.e. a score of 4 or lower) .If the "no” option is used on questions 2, 4, 6 or 8, the review is likely to have major flaws (i.e. a score of 3 or less, depending on the number and degree of the flaws).* | | | **7** |

REF ID: **Sykes M.J., McAnuff J. & Kolehmainen N.** *When is audit and feedback effective in dementia care? A systematic review* (2018)

| **Questions** | | **NO**  Can´t tell or no info | **PARTIALLY**  Incomplete report | **YES** |
| --- | --- | --- | --- | --- |
| **1** | **Were the search methods used to find evidence (primary studies) on the primary question(s) stated?** |  | *Only the search terms are presented* | *Search terms are presented, and it is also explained how these search terms are combined (by “AND”, “OR”, etc.)* |
| **2** | **Was the search for evidence reasonably comprehensive?** |  | *Only Pubmed/Medline has been searched*  *or*  *Search period is shorter than 10 years* |  |
| **3** | **Were the criteria used for deciding which studies to include in the review reported?** |  | *Only exclusion criteria are presented* | *Inclusion criteria are explicitly described* |
| **4** | **Was bias in the selection of articles avoided?** |  | *Only a part of the selection steps was completed by two reviewers independently* | *Both the initial selection (tiltle & abstract) and full-text reading were performed by two reviewers independently* |
| **5** | **Were the criteria used for assessing the validity of the studies that were reviewed reported?** |  |  | *The reviewer(s) used explicit assessment criteria or an existing assessment checklist* |
| **6** | **Was the validity of all of the studies referred to in the text assessed using appropriate criteria (either in selecting studies for inclusion or in analyzing the studies that are cited)?** |  | *Only the rigorousness of research designs is taken into account* | *Both in the selection phase and during analysis the methodological quality of the studies is taken into account using explicit criteria* |
| **7** | **Were the methods used to combine the findings of the relevant studies (to reach a conclusion) reported?** |  |  | *A “best evidence synthesis” is performed*  *Pooling of results has taken place*  *Results are presented on the basis of a an “evidence table”* |
| **8** | **Were the findings of the relevant studies combined appropriately relative to the primary question the review addresses?** | *No attempt made to combine findings*  *No statement regarding the inappropriateness of combining findings*  *A summary (general) estimate is given* *anywhere in the abstract, the discussion or the summary section of the paper but it is not reported how that estimate was derived* |  |  |
| **9** | **Were the conclusions made by the author(s) supported by the data and/ or analysis reported in the review?** |  |  | *Data (not just citations) should be reported that support the main conclusions* |
| **10** | **Overall, how would you rate the scientific quality of this review?**   \| Extensive flaws \|  \| Major flaws \|  \| Minor flaws \|  \| Minimal flaws \| \| --- \| --- \| --- \| --- \| --- \| --- \| --- \| \| 1 \| 2 \| 3 \| 4 \| 5 \| 6 \| 7 \|   *The score for question 10, the overall scientific quality, should be based on your answers to the first nine questions. The following guidelines can be used to assist with deriving a summary score: If the "can't tell" option is used one or more times on the preceding questions, a review is likely to have minor flaws at best and it is difficult to rule out major flaws (i.e. a score of 4 or lower) .If the "no” option is used on questions 2, 4, 6 or 8, the review is likely to have major flaws (i.e. a score of 3 or less, depending on the number and degree of the flaws).* | | | **4** |
